# Plant-Mediated Effects of Water Deficit on the Performance of *Tetranychus evansi* on Tomato Drought-Adapted Accessions

**DOI:** 10.3389/fpls.2018.01490

**Published:** 2018-10-17

**Authors:** Miguel G. Ximénez-Embún, Miguel González-Guzmán, Vicent Arbona, Aurelio Gómez-Cadenas, Félix Ortego, Pedro Castañera

**Affiliations:** ^1^Laboratorio de Interacción Planta-Insecto, Departamento de Biotecnología Microbiana y de Plantas, Centro de Investigaciones Biológicas, CSIC, Madrid, Spain; ^2^Ecofisiologia i Biotecnologia, Departament de Ciències Agràries i del Medi Natural, Universitat Jaume I, Castellón de la Plana, Spain

**Keywords:** plant**-**herbivore interaction, abiotic stress, drought stress, spider mites, Tomàtiga de Ramellet

## Abstract

Climate change is expected to increase drought periods and the performance and dispersal of some invasive species such as *Tetranychus evansi*, which has been reported to take advantage of the nutritional changes induced by water-shortage on the tomato cultivar Moneymaker (MM). We have examined the implications for mite’s biology of four accessions of the drought-adapted tomatoes, “Tomàtiga de Ramellet” (TR), under moderate drought stress. Mite performance was enhanced by drought in two accessions (TR61 and TR154), but not in the other two accessions (TR58 and TR126). We selected one accession of each outcome (i.e., TR154 and TR126) to further analyze plant nutritional parameters. We found that free sugars and most essential amino acids for mites were induced by drought and/or mite infestation on MM and TR154 plants, whereas sugars were not altered and a reduced number of essential amino acids were induced by drought in TR126. Remarkably, mite performance was enhanced by leaf infiltration of free sugars, essential amino acids mixture, and L-proline on well-watered MM and by free sugars on drought-stressed TR126 plants. These results indicate a positive link between the induction of soluble carbohydrates and amino acids used by the plant for osmotic adjustment and mite performance. The effects of drought and/or mite infestation on the defense response of plants was analyzed at three levels: phytohormone accumulation, the transcript levels of marker genes linked to jasmonates (JAs), salicylic acid (SA), and abscisic acid (ABA) pathways, and the activity of defense proteins. The ability of *T. evansi* to downregulate the accumulation of defense-related phytohormones was noted on MM and the two TR accessions analyzed (TR126 and TR154), though differences in the induction of protein defense genes and activities by drought and/or mite infestation were observed among them. These results emphasize the importance of studying plant biotic and abiotic stress factors in combination and provides an experimental framework for screening drought-tolerant tomato accessions that will be also resistant to herbivore mites.

## Introduction

Tomato (*Solanum lycopersicum* L.) is a major vegetable crop grown all over the world in outdoor fields and greenhouses. During cultivation, tomato plants are exposed to a combination of biotic and abiotic stresses, soil water deficiency, and arthropod pests being among the most critical. Its cultivation is mainly concentrated in semiarid zones, like the Mediterranean, where it needs to be cultivated under irrigation (Rivelli et al., 2013), and where drought events associated with climate change are expected to be more frequent (Nankishore and Farrell, 2016). Thus, water shortage caused by drought periods can have important consequences for tomato production, as it might produce yield reduction of up to a 50% in the case of an equivalent reduction in irrigation (Cantore et al., 2016). Tomato plants are attacked by a number of insect and mite pests which significantly reduce fruit yield and quality. Worldwide, losses due to these pests are estimated to be about 34.4% of attainable tomato yield under current production practices (Zalom, 2003). Moreover, the sustainability of tomato production is threatened by an increasing number of invasive arthropod pests (Navajas et al., 2013; Tonnang et al., 2015).

The high sensitivity of tomato to water deficit and the need for irrigation in the Mediterranean basin has prompted different approaches for breeding drought-resistant crops, including the search for drought tolerant/adapted varieties (Hu and Xiong, 2014). The term “drought-adapted” refers to higher yield in crop plants under water shortage conditions (Verslues and Juenger, 2011). In this regard, finding tomato drought-adapted varieties might become an important approach to improve water use efficiency under future climate change scenarios. Hereof, the “Tomàtiga de Ramellet” tomatoes, which represent a population of landraces from the Balearic Islands (Spain), have been traditionally cultivated outdoors under low water availability conditions during the Mediterranean summer, and thus they represent tomatoes adapted to cultivation under water deficit (Galmes et al., 2011, 2013). Several studies have compared groups of tomato varieties, landraces or wild species, observing a differential expression on some drought-associated traits between varieties, but without having a consistent response in the literature. For instance, the osmolite L-proline is usually induced by drought, but the level of induction is a variety-dependent trait, whereas leaf water content is generally less reduced on tolerant plants (Sanchez-Rodriguez et al., 2010; Tapia et al., 2016). The accumulation of osmolites, mainly soluble free sugars and amino acids, which facilitate water uptake and retention, have been reported in tomato leaves during restricted watering (English-Loeb et al., 1997; Tapia et al., 2016). Other traits such as stomatal conductance and the maximum quantum yield of photosystem II photochemistry (Fv/Fm) are parameters commonly used as drought response indicators. In general, a fast decrease in stomatal conductance and a long maintenance of the Fv/Fm value, are indicative of tolerance (Thompson et al., 2007; Mishra et al., 2012; Nankishore and Farrell, 2016; Tapia et al., 2016). To our knowledge, molecular mechanisms driving drought adaptation on the aforementioned drought-adapted landraces are unknown.

The red tomato spider mite, *Tetranychus evansi* Baker and Pritchard, first recorded in Brazil, has emerged as a serious invasive pest in some areas of Africa and Europe (Navajas et al., 2013), because it is highly tolerant to hot and dry conditions. Thus, it is expected to spread northward across Europe (Meynard et al., 2013) under a climate change scenario. Furthermore, it is able to suppress tomato plant defenses (Kant et al., 2015) by downregulating the accumulation of defense-related phytohormones and the expression of genes involved in the regulation of secondary metabolites and defense proteins (Sarmento et al., 2011; Alba et al., 2015; Ataide et al., 2016; Schimmel et al., 2017). In addition, we have found that both drought and *T. evansi* infestation induced significant changes in the nutritional quality of tomato plants of the commercial cultivar, Moneymaker (MM) (Ximénez-Embún et al., 2016). Specifically, we found that more essential amino acids and free sugars were available in leaf tissues under drought conditions, and that L-proline, the amino acid highest induced by drought in tomato, had a phagostimulant effect on *T. evansi*. These physiological changes trigger a bottom-up effect on key biological traits of *T. evansi* causing a highly significant increase in leaf damage and mite performance, thus it represents an increasing threat to tomato crop production in the face of climate change (Ximénez-Embún et al., 2016).

A complex phytohormonal network drives plant responses to biotic and abiotic stresses and it is responsible of fine-tuning plant physiology response to a specific stress (Spoel et al., 2007; Bostock et al., 2014). Plant responses to drought stress are mainly controlled by abscisic acid (ABA) (Cutler et al., 2010), whereas plant responses to biotic stresses are mediated by salicylic acid (SA), jasmonic acid, and derivates named as jamonates (JAs) and ethylene (ET). Plant response to a combination of stresses is a complex trait that cannot be directly deduced from that to each of the different stress applied individually (Atkinson and Urwin, 2012; Suzuki et al., 2014), but both stresses contribute to the final responses (Coolen et al., 2016). However, despite the wealth of sources of variation for drought tolerance in accessions of tomato and wild-related species, the mechanisms that govern their responses to water stress are not well characterized (Egea et al., 2018). Moreover, the consequences that these mechanisms may impose on their interaction with biotic stresses are largely unknown. We report here a holistic approach that considers both drought and spider mites. Accordingly, we investigate the performance of *T. evansi* on drought-adapted “Tomàtiga de Ramellet” tomatoes subjected to a moderate water deficit. The mite performance response when feeding on some of the selected accessions was linked to the observed changes in plant nutritional composition and plant defense responses as compared to the commercial cultivar MM that we have used as model in previous works (Ximénez-Embún et al., 2016). Furthermore, by imposing these two stresses in combination, the possible directions of the multiple physiological interactions could be examined.

## Materials and Methods

### Plant Material and Mite Rearing

Four tomato accessions (provided by Dr. Granell, IBMCP, Valencia, Spain) were used in this study: TR58, TR61, TR126, and TR154 (from a population of local landraces “Tomàtiga de Ramellet” (TR) from Mallorca, Balearic Islands, Spain) traditionally grown under non-irrigated conditions during the Mediterranean summer and therefore adapted to water deficit conditions; as well as the commercial cultivar MM. Tomato plants were grown from seeds in 40-well trays. Plants with three expanded leaves were transferred to 2.5 l pots (diameter: 16 cm, height: 15 cm) (Maceflor, Valencia, Spain) filled with 600 g of Universal growing medium “Compo sana^®^” (Compo GmbH, Münster, Germany) and watered to saturation.

A colony of *T. evansi* derived from the Nice strain collected in Beausoleil (South of France) was provided by Dr. Maria Navajas (CBGP, France). Mites were maintained on detached MM tomato leaves placed on ventilated plastic cages (22 cm × 30 cm × 15 cm) for about 50 generations. Inside the cages, leaves were placed on a plastic plate that was on top of a soaked sponge. The petioles of the leaves were in contact with a thin layer of water in the bottom of the cages to maintain the leaf turgor and to contain the mites.

Plants and mite cages were maintained in climate rooms at 25 ± 1°C, 50 ± 5% relative humidity and a 16 h light/8 h dark photoperiod.

### Drought Stress Regime

Drought stress was attained by water deficiency as described by Ximénez-Embún et al. (2016). In brief, tomato plants were well-watered until they developed four to five fully expanded leaves, then we imposed two irrigation regimes, defined as control and moderate drought stress. Control plants were watered regularly to maintain the soil volumetric water content (𝜃) up to 74%. For moderate drought stress, watering was stopped for 7 days and thereafter plants were watered to maintain 𝜃 between 21 and 30%. Steady stress conditions were reached at about 7–9 days after ceasing irrigation. Water-stressed plants from the four accessions were over the wilting point associated with severe drought stress, established at 𝜃 ≤ 16% for MM in our experimental conditions (Ximénez-Embún et al., 2016). 𝜃 was determined gravimetrically by recording single plant pot weight (balance BSH 6000, PCE Iberica, Tobarra, Spain).

The severity of drought stress was assessed by measuring the following parameters on the sub-terminal leaflet of the fourth leaf: (a) stomatal conductance (gs) using a leaf porometer (SC-1 Decagon-T, Pullman, WA, United States); and (b) variations in maximum quantum yield of photosystem II photochemistry (Fv/Fm), using a FluorPen FP 100 (PSI, Drasov, Czech Republic). Plant growth was estimated by measuring the stem length (distance between the soil and the terminal bud).

### Bioassays

Three different experiments were carried out: (1) to measure the effect of moderate drought on mite performance and leaf damage; (2) to determine the effects of moderate drought and mite infestation on tomato nutritional composition and plant defenses; and (3) to test the stimulatory effect of free sugars and amino acids in mite performance. All experiments were carried out in a climate room under the same environmental conditions, as those described above for mite rearing.

#### Experiment 1

Effect of drought on mite performance and leaf damage. Tomato plants from each of the four TR accessions were randomly assigned to control or moderate drought treatments. At about 7–9 days, after stopping irrigation, the drought stress conditions had stabilized, corresponding with the phenological stage of six to seven expanded tomato leaves. Then, plants were infested with *T. evansi* females of random age collected from the laboratory colony by using a vacuum pump D-95 (Dinko S.A., Barcelona, Spain) with a sucking power of 10–50 mmHg connected to a modified polypropylene microtube. They were placed on the two sub-terminal leaflets of tomato leaves three, four, and five (eight mites per leaflet, 48 mites per plant). All plants (infested and non-infested) were confined with a ventilated metacrylate cylinder, fitting the pot diameter, to avoid mites escaping from the infested plants and to simulate similar environmental conditions in the non-infested ones. They were set up in a climate room following a complete randomized block design. A total of nine replicates per treatment were simultaneously performed. Mite performance was assessed at 4 days post infestation (dpi) to avoid overlapping generations, as eggs need at least 5 days to hatch in our experimental conditions. All leaves were detached from the plants, and the number of eggs and mobile mite stages (larvae, nymphs, and adults) were counted under a stereomicroscope M125 (Leica Mycrosystem, Wetzlar, Germany). The leaf damaged area (mm^2^ of chlorotic lesions) was determined by scanning the damaged leaflets using hp scanjet (HP Scanjet 5590 Digital Flatbed Scanner series, United States) and analyzing the scanned leaflets with the program GIMP 2.8^1^, as described in Ximénez-Embún et al. (2016).

#### Experiment 2

Effect of drought and *T. evansi* on plant nutritional composition and plant defenses. Plants of the accessions TR126 and TR154 and of the cultivar MM were assigned to four different groups combining two treatments: uninfested well-watered plants (Control); uninfested drought-stressed plants (Drought); infested well-watered plants (*T. evansi*); and uninfested drought-stressed plants (Drought + *T. evansi*). When drought stress conditions had stabilized, plants were infested as described above. Four days after infestation, plant material was collected. The left leaflets from leaves three, four, and five were pooled, ground in liquid nitrogen to a fine powder, and stored at -80°C for the analysis of total protein, free amino acids, and plant defense responses (phytohormone accumulation, transcript levels of stress marker genes, and enzymatic activity of defense proteins). The right leaflets from the same leaves were pooled and immediately weighed, oven-dried at 70°C for 3 days and weighed again to assess the percentage of water, ground using a mortar and pestle to obtain a fine powder, and stored at room temperature for free sugar analysis. Six replicates per treatment were used.

#### Experiment 3

Stimulatory effect of free sugars and amino acids in mite performance. Tomato leaves of the cultivar MM and the accession TR126 were infiltrated with solutions of sugars, essential amino acids, and L-proline. The accession TR126 was chosen as mite performance on it differs to the observed on MM, and we wanted to test whether this difference remains when infiltrating nutrients. Concentrations were chosen to simulate their induction by both drought and *T evansi* infestation on tomato plants of the cultivar MM at 4 dpi (see Section “Results” “Changes in Plant Nutritional Composition Induced by Drought and *T. evansi*”), corresponding to 38 mg of free sugars, 9.66 mg of essential amino acids, and 2.42 mg of L-proline/g of leaf dry weight, respectively. Accordingly, a solution of essential amino acids containing L-valine (0.39 g/l), L-isoleucine (0.29 g/l), L-leucine (0.27 g/l), L-tyrosine (0.27 g/l), L-phenylalanine (0.21 g/l), L-histidine (0.19 g/l), and L-arginine (0.83 g/l) was prepared, based on their relative proportions in the tomato leaves (see **Table 1**) and an estimated fresh leaflet weight of 0.5 g and a ratio of 0.06 dry/fresh leaflet weight. In the case of L-proline, its concentration in the solution was 0.61 g/l. For the free sugars, a solution including sucrose (4.62 g/l), glucose (2.78 g/l), and fructose (1.85 g/l) was prepared, based on the total amount of free sugars (see **Figure 2**) and the relative amount of these sugars in tomato leaves as reported by Khodakovskaya et al. (2010). Both, control and moderate drought-stressed plants were infiltrated, with five replicates per treatment. The infiltration protocol was as follows: the sub terminal leaflet of the third leaf was infiltrated with approximately 0.2 ml of an aqueous solution using a 1 ml needleless syringe. It was allowed to dry for 1 h and then 100 females of *T. evansi* were placed on the infiltrated leaflet. A barrier of Lanolin (Manuel Riesgo, Madrid, Spain) was placed on the petiolule of the leaflet to avoid the mites to escape. After 24 h, the number of eggs per leaflet was determined. To measure the incorporated free amino acids and sugars into the tomato leaf by infiltration, MM leaves were infiltrated but not infested with mites and leaflets were collected at 1 h post infiltration (hpi), dried at 70°C for 3 days, ground using a mortar and pestle to obtain a fine powder for the analysis of free sugars and L-proline.

**Table 1:** Effect of moderate drought stress, *T. evansi* infestation, and their combination (Dr + Te) on the amino acid composition of the tomato accessions TR126, TR154, and the cultivar Moneymaker at 4 days post infestation.

	TR126					TR154					Moneymaker				
	Control	Drought	*T. evansi*	Dr + Te	SF	Control	Drought	*T. evansi*	Dr + Te	SF	Control	Drought	*T. evansi*	Dr + Te	SF
Non-essential amino acids							
Asp	2.10 ± 0.50	2.79 ± 0.91	2.97 ± 0.52	1.91 ± 0.18	–	4.00 ± 0.89	2.02 ± 0.17	2.84 ± 1.12	3.80 ± 0.83	–	1.47 ± 0.11	2.57 ± 1.28	1.32 ± 0.08	3.04 ± 0.80	–
Thr	0.40 ± 0.10	0.98 ± 0.36	0.58 ± 0.09	0.71 ± 0.08	D	1.10 ± 0.28	0.89 ± 0.11	0.78 ± 0.27	1.48 ± 0.27	–	0.54 ± 0.07	1.70 ± 0.94	0.57 ± 0.03	1.83 ± 0.37	D
Ser	2.00 ± 0.70	2.68 ± 1.02	2.77 ± 0.92	2.72 ± 0.51	–	2.49 ± 0.45	4.42 ± 0.97	2.44 ± 0.62	7.36 ± 1.53	D	1.87 ± 0.43	8.42 ± 5.52	1.80 ± 0.26	11.8 ± 3.41	D
Glu	3.20 ± 0.40a	10.00 ± 3.60b	4.79 ± 0.25ab	5.13 ± 0.90ab	D,I	12.00 ± 2.60a	7.33 ± 0.52a	8.82 ± 2.63a	14.09 ± 3.00a	I	4.21 ± 0.50a	4.34 ± 1.00a	4.34 ± 0.30a	8.38 ± 1.15b	D,M,I
Gly	0.03 ± 0.01	0.05 ± 0.02	0.04 ± 0.01	0.07 ± 0.03	–	0.04 ± 0.01	0.03 ± 0.01	0.03 ± 0.01	0.04 ± 0.01	–	0.02 ± 0.01	0.13 ± 0.11	0.02 ± 0.01	0.05 ± 0.01	–
Ala	0.35 ± 0.09	0.43 ± 0.16	0.43 ± 0.08	0.49 ± 0.21	–	0.41 ± 0.12	0.26 ± 0.04	0.28 ± 0.09	0.47 ± 0.10	–	0.22 ± 0.06	0.92 ± 0.73	0.25 ± 0.06	0.56 ± 0.13	–
Cys	0.03 ± 0.01	0.05 ± 0.02	0.04 ± 0.01	0.05 ± 0.01	–	0.06 ± 0.01	0.05 ± 0.01	0.05 ± 0.02	0.10 ± 0.02	–	0.02 ± 0.01	0.12 ± 0.1	0.02 ± 0.01	0.09 ± 0.02	D
Pro	0.08 ± 0.02	0.79 ± 0.27	0.13 ± 0.02	0.84 ± 0.32	D	0.28 ± 0.06	0.75 ± 0.09	0.2 ± 0.05	1.39 ± 0.44	D	0.11 ± 0.01	1.20 ± 0.79	0.11 ± 0.01	2.42 ± 0.75	D
Essential amino acids							
Val	0.16 ± 0.04	0.36 ± 0.13	0.22 ± 0.04	0.51 ± 0.16	D	0.36 ± 0.07	0.65 ± 0.17	0.41 ± 0.1	1.10 ± 0.22	D	0.26 ± 0.08	1.55 ± 1.16	0.29 ± 0.04	1.55 ± 0.41	D
Met	0.02 ± 0.01	0.03 ± 0.02	0.03 ± 0.01	0.08 ± 0.05	–	0.04 ± 0.01	0.03 ± 0.01	0.03 ± 0.01	0.04 ± 0.01	–	0.02 ± 0.01	0.21 ± 0.18	0.02 ± 0.01	0.07 ± 0.02	–
Ile	0.10 ± 0.02	0.20 ± 0.07	0.15 ± 0.04	0.33 ± 0.10	D	0.21 ± 0.04	0.47 ± 0.14	0.28 ± 0.08	0.80 ± 0.18	D	0.18 ± 0.05	1.01 ± 0.75	0.21 ± 0.04	1.38 ± 0.24	D
Leu	0.08 ± 0.02	0.14 ± 0.05	0.11 ± 0.03	0.46 ± 0.25	D	0.20 ± 0.04	0.38 ± 0.13	0.25 ± 0.06	0.65 ± 0.14	D	0.15 ± 0.05	1.45 ± 1.23	0.18 ± 0.03	1.32 ± 0.24	D
Tyr	0.07 ± 0.02	0.1 ± 0.03	0.11 ± 0.03	0.27 ± 0.12	–	0.13 ± 0.02	0.36 ± 0.13	0.22 ± 0.07	0.55 ± 0.12	D	0.13 ± 0.05	0.86 ± 0.66	0.16 ± 0.03	1.46 ± 0.22	D
Phe	0.13 ± 0.02	0.26 ± 0.09	0.19 ± 0.02	0.42 ± 0.17	D	0.34 ± 0.07	0.35 ± 0.09	0.34 ± 0.10	0.62 ± 0.12	–	0.19 ± 0.03	1.04 ± 0.82	0.2 ± 0.02	0.84 ± 0.20	D
His	0.06 ± 0.02	0.09 ± 0.03	0.10 ± 0.04	0.15 ± 0.04	–	0.11 ± 0.02	0.33 ± 0.08	0.18 ± 0.04	0.68 ± 0.19	D	0.12 ± 0.04	0.45 ± 0.28	0.13 ± 0.03	0.88 ± 0.16	D
Lys	0.09 ± 0.03	0.12 ± 0.05	0.15 ± 0.06	0.37 ± 0.20	–	0.17 ± 0.03	0.36 ± 0.09	0.19 ± 0.05	0.63 ± 0.12	D	0.11 ± 0.03	1.17 ± 0.97	0.13 ± 0.02	1.09 ± 0.24	D
Arg	0.30 ± 0.13	0.07 ± 0.04	0.73 ± 0.55	0.24 ± 0.12	–	0.16 ± 0.03	0.40 ± 0.15	0.27 ± 0.10	0.63 ± 0.15	D	0.15 ± 0.05	2.10 ± 1.64	0.18 ± 0.04	3.93 ± 1.33	D


*Data are the mean amount of amino acid (mg) per gram of dry weight ± SE of six replicates/treatment. The division between essential and non-essential amino acids is based on a study with the closely related species *Tetranychus urticae* (Rodriguez and Hampton, 1966). Significant factors (SF) indicates whether either of the two independent factors D (drought condition) and M (mite infestation) and/or their interaction I (D × M) are statistically significant for a given cultivar/accession (two-way ANOVA, *p* < 0.05). When the interaction D × M was significant, Newman-Keuls *post hoc* test were performed (different lowercase letters indicate significant differences among treatments). The detailed results of the two-way ANOVA (*F* and *p* values and degrees of freedom) are shown in **Supplementary Tables S6–S8***.

### Chemical and Biochemical Analysis

#### Chemicals and Equipment

Unless specified otherwise, all chemical compounds were obtained from Sigma-Aldrich (St Louis, MO, United States). Fluorimetric measurements were made using a Varioskan Flash reader (Thermo Fisher Scientific, Wilmington, DE, United States), and spectrophotometric measurements with a VERSAmax microplate reader (Molecular Devices Corp., Sunnyvale, CA, United States).

#### Free Sugars

Samples of 40 mg of leaf powder were homogenized in 650 μl of 95% (v/v) aqueous ethanol and heated at 80°C for 20 min. Samples were then centrifuged at 10,000 rpm for 10 min, and the supernatant collected. The process was repeated two more times and the three supernatants were pooled. A volume of 750 μl of the mixture was dried on a SpeedVac Concentrator Savant SVC-100H (Thermo Fisher Scientific) and redissolved in 500 μl of water. Soluble carbohydrate concentration was estimated by the anthrone method (Maness, 2010) using glucose as standard. In brief, 1 ml of anthrone reagent (0.2% v/v anthrone on 95% sulfuric acid) was added to the extract, heated at 90°C for 15 min, and the absorbance measured at 630 nm.

#### Free Amino Acids

The extraction of free amino acids was done as described by Hacham et al. (2002). Samples of 50 mg of frozen leaf powder were homogenized with 600 μl of water:chloroform:methanol (3:5:12 v/v/v). After centrifugation at 12,000 rpm for 2 min, the supernatant was collected and the residue was re-extracted with 600 μl of the same mixture, pooling the two supernatants. A mixture of 300 μl of chloroform and 450 μl of water were added to the supernatants, and after centrifugation the upper water:methanol phase was collected and dried in the SpeedVac. The samples were dissolved on 100 μl of sodium citrate loading buffer pH 2.2 (Biochrom, United States) and 10 μl were injected on a Biochrom 30 Amino Acid Analyser (Biochrom, United States) at the Protein Chemistry Service at CIB (CSIC, Madrid, Spain).

#### Soluble Protein

Samples of 100 mg of leaf frozen powder were homogenized in 500 μl of 0.15 M NaCl and ground with fine sand. The homogenate was centrifuged at 12,000 rpm for 5 min at 4°C, and the soluble protein quantified by absorbance at 280 nm on a Nanodrop 2000 spectrophotometer (Thermo Fisher Scientific, Wilmington, DE, United States).

#### Plant Defense Proteins

Samples of 100 mg of leaf frozen powder were homogenized with 500 μl of extraction buffer (0.15 M NaCl for protease inhibitors, and 0.1 M phosphate buffer, pH 7.0; 5% w:v polyvinylpolypyrrolidine for oxidative enzymes) and soluble protein quantified as explained above.

The inhibitory activity of plant protein extracts was tested against commercial enzymes: papain (EC 3.4.22.2), cathepsin B from bovine spleen (EC 3.4.22.1), trypsin from bovine pancreas (EC 3.4.21.4), α-chymotrypsin from bovine pancreas (EC 3.4.21.1), cathepsin D from bovine spleen (EC 3.4.23.5), and leucine aminopeptidase from porcine pancreas (EC 3.4.11.1), as described by Ximénez-Embún et al. (2016). Reaction conditions are summarized in **Supplementary Table S1**. Results were expressed as a percentage of protease activity inhibited.

Polyphenol oxidase (PPO) activity was analyzed by incubating 20 μl of enzyme extract with cathecol (40 mM final concentration) in 160 μl of Tris-HCl pH 8.5 buffer at 30°C for 1 h. Absorbance was read at 420 nm. Peroxidase (POD) activity was determined incubating 20 μl of a 1:10 dilution of the enzyme extract with guaiacol (5 mM final concentration) and H_2_O_2_ (2.5 mM final concentration) in 150 μl of potassium phosphate pH 6 buffer at 30°C for 10 min. Absorbance was read at 470 nm. PPO and POD activities were expressed as nmol substrate metabolized relative to time and total protein content.

#### L-Proline Analysis

The L-proline content of infiltrated leaves was analyzed adapting the protocol described by Bates et al. (1972). Samples of 4 mg of dry leaf powder were homogenized in 1 mL of a solution of sulfosalicylic acid 3% (w/v). After centrifugation at 10,000 rpm for 10 min, the supernatant was collected. A mix of 250 μl of supernatant, 250 μl of glacial acetic acid (AcH) and 250 μl of acid ninhydrin reagent [acid ninhydrin 2.5% (w/v), AcH 60% (v/v), H_3_PO_4_ 2.5 M] was incubated for 1 h at 100°C. The reaction was stopped in an ice bath for 10 min. The products of the reactions were extracted by adding 500 μl of toluene and mixing vigorously for 15-20 s. The absorbance of the aqueous supernatant was measure at 520 nm. The L-proline concentration was determined from a commercial L-proline standard curve.

### Quantification of Phytohormones

Freeze-dried plant material (c.a. 10 mg) was spiked with 25 μl of an internal standard mixture (containing ABA-*d_6_*, DHJA, and ^13^C_6_-SA) to correct for analyte losses and extracted in 1 mL ultrapure water for 10 min in a ball mill at room temperature. After extraction, homogenates were centrifuged at 10,000 rpm for 10 min at 4°C to remove debris. Supernatants were recovered, pH adjusted to 3.0 with 30% acetic acid, and partitioned twice against an equal volume of di-ethyl ether. The combined organic layers were evaporated under vacuum in a centrifuge concentrator (Jouan, Saint Germain Cedex, France). The dry residues were subsequently reconstituted in 0.5 mL of 10% aqueous methanol and the resulting solutions filtered through 0.20 μm syringe membrane filters. Filtered extracts were analyzed by tandem LC/MS in an Acquity SDS UPLC system (Waters Corp., United States) coupled to a TQS triple quadrupole mass spectrometer (Micromass Ltd., United Kingdom) through an electrospray ionization source. Separations were carried out on a C18 column (Gravity C18, 50 mm × 2.1 mm, 1.8 μm particle size, Macherey-Nagel, Germany) using a linear gradient of ultrapure methanol and water, both supplemented with acetic acid to a 0.1% concentration, at a constant flow rate of 0.3 mL.min^-1^. During analyses, column was maintained at 40°C and samples at 10°C to slow down degradation. Plant hormones were detected in negative electrospray mode by their specific precursor-to-product ion transitions (ABA, 263 > 153; JA, 209 > 59; SA, 137 > 93; 12-oxo-phytodienoic acid OPDA, 291 > 165; and SA glucosyl ester SAGE 299 > 137) and quantitated using an external calibration curve with standards of known amount.

### Quantification of Gene Expression *via* qRT-PCR

The expression levels of drought response *RAB18* gene, SA-dependent *PR1a* gene, and JA-dependent *MYC2*, *CDI*, *PPO-F*, and *PI-Ia* genes were measured by qRT-PCR. Samples of 100 mg of frozen leaf powder were taken for total RNA extraction using the TRIZOL Reagent (Molecular Research Center, Cincinnati, OH, United States) according to the manufacturer’s instructions. RNA quantification was done using NanoDrop 2000 spectrophotometer (NanoDrop Technologies Inc., Wilmington, DE, United States). Two micrograms of total RNA previously treated with RQ1 DNase (Promega, Madison, WI, United States) during 35 min at 37°C followed by 10 min at 75°C was used for single strand cDNA synthesis. Reverse transcription was carried out using the Revert Aid H Minus First Strand cDNA Synthesis Kit (k1632, Thermo Fisher Scientific) according to the manufacturer’s instructions and with some minor modifications. qPCR was carried out in a Corbett Rotor Gene 6000 real-time cycler (Qiagen) using the Brilliant III Ultra-Fast SYBR Green QPCR Master Mix (Agilent Technologies, Santa Clara, CA, United States) as previously described (Vidal-Quist et al., 2015). Raw gene expression data were efficiency-corrected and gene expression was transformed to normalized relative quantities (NQR) by using the reference genes (Hellemans et al., 2007). The primers used are listed in **Supplementary Table S2**.

### Statistical Analysis

All plant and mite data were checked for the assumptions of normality and heteroscedasticity, and transformed if necessary. Stem length, stomatal conductance, *T. evansi* eggs and mobile forms, leaf damaged area, and phytohormone data were log_10_(*x*) transformed, Fv/Fm was log_10_(*x* + 1) transformed; gene-expression data (NRQ values) were ln(*x* + 1) transformed; and the percentage of water, free sugars, protein, total free amino acids, and protease inhibition activities were arcsine square root transformed. Different types of statistical analysis were performed depending on the design and purpose of each experiment. Student’s *t*-test were performed to determine whether moderate drought has an effect on plant functional traits (stomatal conductance, Fv/Fm, and stem length) and mite performance (*T. evansi* eggs and mobile forms, and leaf damaged area) for each plant cultivar/accession tested (Experiment 1). Two-way ANOVA were performed to test the effect of drought condition (D), mite infestation (M), and their combination on plant nutritional parameters (percentage of water, free sugars, protein and free amino acids, and amount of specific amino acids) and plant defenses (phytohormone levels, gene-expression, protease inhibition, and oxidative enzyme activities) for a given cultivar/accession (Experiment 2). When the interaction D × M was significant, Newman-Keuls *post hoc* test were performed to compare treatments. The infestation by *T. evansi* did not affect plant functional traits (stomatal conductance, Fv/Fm, and stem length) in Experiment 2, and thus, data from infested and non-infested plants were pooled and re-analyzed also by Student’s *t*-test for each plant cultivar/accession tested. One-way ANOVA followed by Newman-Keuls *post hoc test* was performed to compare the effect of the agroinfiltration of leaflets with different nutrients on mite performance (number of eggs) and the content of free sugars and L-proline recovered from infiltrated leaflets at 1 and 24 h post-infiltration (Experiment 3). The correlation between the levels of plant nutrients (free sugars and L-proline) and the number of mite’s eggs in infiltration experiments was analyzed by using the Pearson’s correlation coefficient. For the statistical analysis, the IBM SPSS Statistics 24.0 software (Chicago, IL, United States) was used.

## Results

### Effect of Drought on Stomatal Conductance, Photosynthetic Efficiency, and Tomato Plant Growth

The effect of drought stress on stomatal conductance, photosynthetic efficiency, and stem length observed in Experiment 1 (**Supplementary Figure S1** and **Supplementary Table S3**) and Experiment 2 (**Supplementary Figure S2** and **Supplementary Table S4**) indicates that the severity of drought stress attained under our experimental conditions can be considered as moderate. In all plant cultivar/accessions, drought induced a significant reduction of stomatal conductance that was, on average, about 3.5 and 4.5 times lower at mite infestation time and at 4 dpi, respectively (**Supplementary Figures S1A**, **S2A**). The maximum efficiency of PSII (Fv/Fm) was not affected by drought in any cultivar/accession, except for a significant increase at mite infestation for TR126 in Experiment 2 (**Supplementary Figures S1B**, **S2B**). Moreover, the Fv/Fm values were never below 0.7, corroborating that severe drought stress conditions were never reached (Ritchie, 2006; Mishra et al., 2012). Moderate drought stress reduced plant growth, as stem length was smaller on drought-stressed plants at the time of infestation for TR58 and TR61 in Experiment 1 and for MM and TR154 in Experiment 2, and in all cultivar/accessions at 4 dpi in both experiments (**Supplementary Figures S1C** **S2C**).

### Effect of Drought on *T. evansi* Performance

**FIGURE 1:**
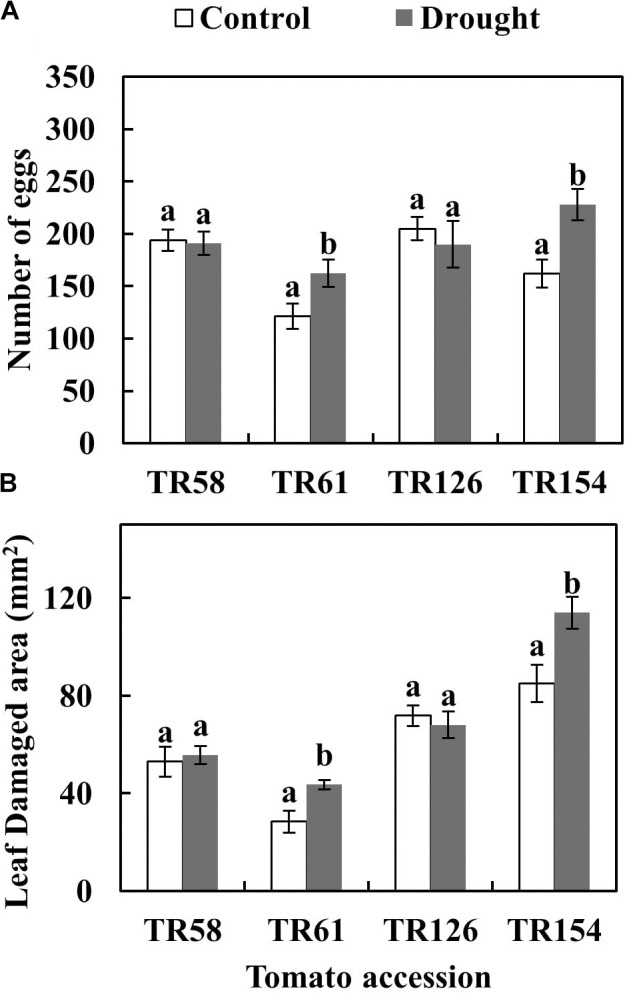
Performance of *T. evansi* in the tomato accessions TR58, TR61, TR126, and TR154. The number of eggs **(A)** and leaf damaged area **(B)** on well-watered (Control) and moderate drought-stressed (Drought) tomato plants at 4 days post infestation were measured. Data are mean ± SE of nine replicates/treatment. Different lowercase letters indicate significant differences within each cultivar/accession (Student’s *t*-test, *p* < 0.05). The detailed results (*t* and *p* values and degrees of freedom) are shown in **Supplementary Table S5**.

A differential effect of moderate drought was observed on *T. evansi* performance depending on the tomato TR accessions on which they were feeding (**Figure 1** and **Supplementary Table S5**). The number of eggs laid by females on drought-stressed plants was 1.3- and 1.4-fold higher than on control plants when feeding on TR61 and TR154, respectively (**Figure 1A**). Likewise, 1.5- and 1.3-fold more damaged area was measured on drought-stressed than on control plants when feeding on TR61 and TR154, respectively (**Figure 1B**). By contrast, similar numbers of eggs and damaged area values were obtained in control and drought-stressed plants when *T. evansi* fed on TR58 and TR126. The number of mobile forms recovered at 4 dpi (surviving females, since eggs need at least 5 days to hatch in our experimental conditions) was not significantly different (**Supplementary Table S5**) between water-stressed and control plants for all TR accessions (data not shown).

The accessions TR154 and TR126 were selected for further chemical, biochemical, and molecular analyses. The rationale for this selection is that they represent the two types of plant-mediated effects of water deficit on mite performance: enhanced (TR154) and no effect (TR126); and that in both cases, the biological parameters obtained on control plants are closer to the values obtained on MM under identical experimental conditions (Ximénez-Embún et al., 2016 observed a 180/341 eggs and 107/133 mm^2^ of damaged area on control/moderate drought-stressed plants, respectively).

### Changes in Plant Nutritional Composition Induced by Drought and *T. evansi*

**FIGURE 2:**
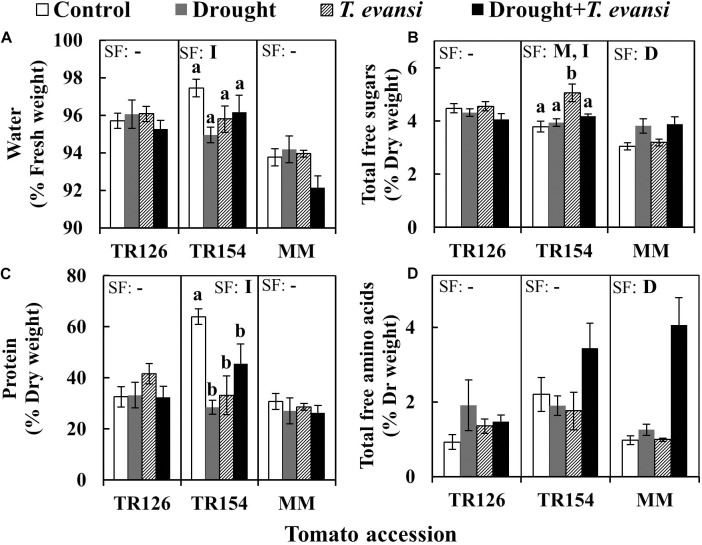
Effect of moderate drought, *T. evansi* infestation and their combination on nutritional composition of tomato accessions TR126 and TR154 and the cultivar Moneymaker (MM) at 4 days post infestation: **(A)** water, **(B)** total free sugars, **(C)** protein, and **(D)** total free amino acids. Data are mean ± SE of six replicates/treatment: uninfested well-watered plants (Control); uninfested drought-stressed plants (Drought); infested well-watered plants (*T. evansi*); and uninfested drought-stressed plants (Drought + *T. evansi*). Significant factors (SF) indicates whether either of the two independent factors D (drought condition) and M (mite infestation) and/or their interaction I (D × M) are statistically significant for a given cultivar/accession (two-way ANOVA, *p* < 0.05). When the interaction D × M was significant, Newman-Keuls *post hoc* test were performed (different lowercase letters indicate significant differences among treatments). The detailed results of the two-way ANOVA (*F* and *p* values and degrees of freedom) are shown in **Supplementary Tables S6–S8**.

The nutritional composition of tomato leaves was determined by analyzing water, free sugars, protein, and total free amino acid content (**Figure 2** and **Supplementary Tables S6–S8**). Drought stress was the most significant factor for MM, inducing the amount of total free sugars and amino acids, whereas *T. evansi* infestation had no significant effect (**Figures 2B,D**). TR154 was affected by *T. evansi* infestation that induced an increase on free sugars, though not when combined with drought stress, and all treatments had lower levels of protein than the control (**Figures 2B,C**). By contrast, drought and/or *T. evansi* infestation did not cause any significant change in the nutritional composition of TR126.

The levels of specific free amino acids, classified as essential or non-essential for the related species *Tetranychus urticae* according to Rodriguez and Hampton (1966), were analyzed (**Table 1** and **Supplementary Tables S6–S8**). L-Proline, an indicator of drought stress, was induced in the three cultivar/accessions by drought. With respect to the rest of amino acids, similar results were obtained with TR154 and MM, which responded to drought stress with an increase of most of the essential amino acids (valine, isoleucine, leucine, tyrosine, histidine, lysine, and arginine) and serine. An increase of threonine, cysteine, and the essential amino acid phenylalanine was also induced by drought in MM, but not in TR154. A comparatively reduced number of essential amino acids (valine, isoleucine, leucine, and phenylalanine) and threonine were induced in TR126 by drought. Mite infestation was only a significant factor for glutamic acid in the case of MM. In addition, a significant interaction between the two factors occurred for glutamic acid in the three varieties/accessions. Thus, the levels of glutamic acid increased under drought conditions but not when combined with mite infestation in TR126, only when both stresses were combined in MM, and no differences among treatments were found in the case of TR154.

### Effect of Drought and *T. evansi* on Tomato Hormones and Stress Response Genes and Defense Proteins

**FIGURE 3:**
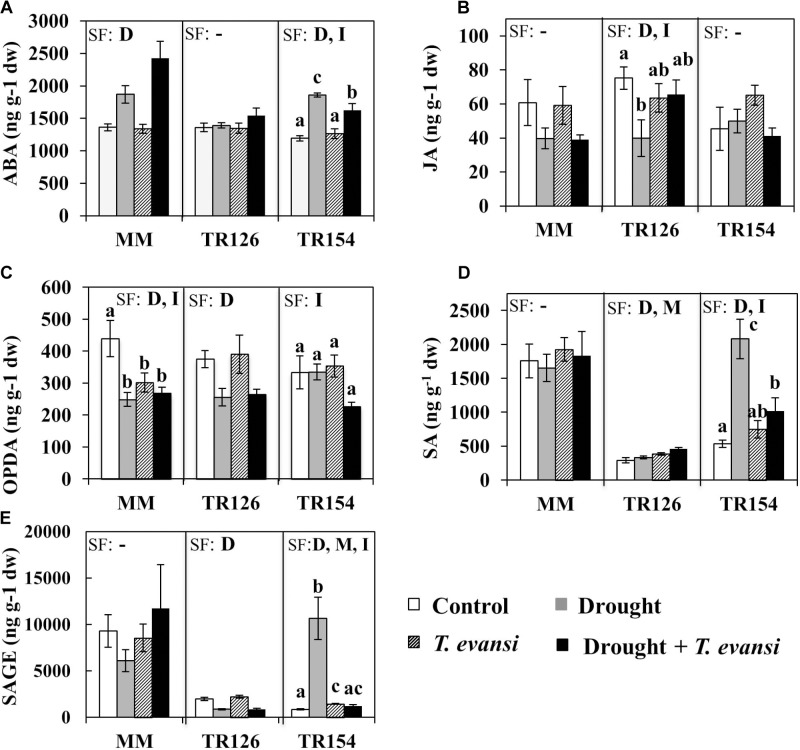
Hormone profiling of tomato accessions TR126 and TR154 and the cultivar Moneymaker (MM) under moderate drought, *T. evansi* infestation, and their combination. Levels of phytohormones **(A)** ABA, **(B)** JA, **(C)** OPDA, **(D)** SA, **(E)** and SAGE were determined at 4 days post infestation. Data are mean ± SE of six replicates/treatment: uninfested well-watered plants (Control); uninfested drought-stressed plants (Drought); infested well-watered plants (*T. evansi*); and uninfested drought-stressed plants (Drought + *T. evansi*). Significant factors (SF) indicates whether either of the two independent factors D (drought condition) and M (mite infestation) and/or their interaction I (D × M) are statistically significant for a given cultivar/accession (two-way ANOVA, *p* < 0.05). When the interaction D × M was significant, Newman-Keuls *post hoc* test were performed (different lowercase letters indicate significant differences among treatments). The detailed results of the two-way ANOVA (*F* and *p* values and degrees of freedom) are shown in **Supplementary Tables S6–S8**.

Hormone profiling in leaf tissues of MM and TR accessions under moderate drought, *T. evansi* infestation, and their combination were determined (**Figure 3** and **Supplementary Tables S6–S8**). As expected, ABA levels in MM and TR154 tomato plants were induced by drought stress, though in the case of TR154 these levels were significantly higher in uninfested drought-stressed plants than in infested drought-stressed plants (**Figure 3A**). On the contrary, TR126 did not show any significant increase in ABA levels, indicating that their molecular response to drought stress is different from that of MM or TR154. The levels of JA were not significantly affected by any stress condition on MM or TR154, whereas a significant reduction of JA levels occurred on uninfested drought-stressed TR126 plants (**Figure 3B**). OPDA levels were reduced in MM in response to all stress conditions and in TR126 in response to drought stress, whereas no significant changes on TR154 could be observed (**Figure 3C**). Interestingly, levels of SA and SAGE in TR126 and TR154 were threefold to fivefold lower than in MM, when plants were maintained under control conditions (**Figures 3D,E**). However, their levels were not significantly affected by any stress condition on MM, whereas (i) both drought stress and mite infestation induced accumulation of SA and drought stress reduced SAGE levels in TR126 and (ii) SA was induced by drought in TR154, but to a lower extent in infested drought-stressed plants, and SAGE was induced by drought stress and mite infestation, but not when both stresses were combined.

**FIGURE 4:**
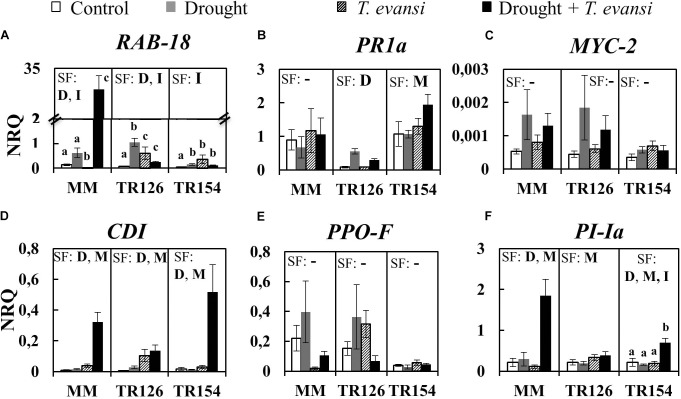
Stress response gene expression analysis of tomato accessions TR126 and TR154 and the cultivar Moneymaker (MM) under moderate drought, *T. evansi* infestation, and their combination. The expression of drought response **(A)** *RAB18* gene, SA-dependent **(B)** *PR1a* gene, and JA-dependent **(C)** *MYC2*, **(D)** *CDI*, **(E)** *PPO-F*, **(F)** and *PI-Ia* genes was analyzed at 4 days post infestation. Data are mean ± SE of six replicates/treatment: uninfested well-watered plants (Control); uninfested drought-stressed plants (Drought); infested well-watered plants (*T. evansi*); and uninfested drought-stressed plants (Drought + *T. evansi*). Significant factors (SF) indicates whether either of the two independent factors D (drought condition) and M (mite infestation) and/or their interaction I (D × M) are statistically significant for a given cultivar/accession (two-way ANOVA, *p* < 0.05). When the interaction D × M was significant, Newman-Keuls *post hoc* test were performed (different lowercase letters indicate significant differences among treatments). The detailed results of the two-way ANOVA (*F* and *p* values and degrees of freedom) are shown in **Supplementary Tables S6–S8**.

Concerning the expression of stress marker genes, the analysis shows different hormone-dependent stress responses in TR accessions and MM (**Figure 4** and **Supplementary Tables S6–S8**). The drought responsive gene *RAB18* was overexpressed under drought and/or in combination of drought and *T. evansi*. This later interaction was especially relevant in the case of MM. However, under *T. evansi* infestation alone, MM and TR accessions showed an opposite pattern, indicating a putative differential ABA-dependent response (**Figure 4A**). The SA-dependent *PR1a* gene expression was significantly induced by drought in TR126 and by mite infestation in TR154, but not in MM (**Figure 4B**). We found also differences in the expression of *CDI* and *PI-Ia* genes, which encode enzymatic activities putatively involved in the response to mite infestation that are under the control of JAs. Thus, *CDI* was overexpressed in response to both drought stress and mite infestation in all varieties/accessions (**Figure 4D**). On the contrary, *PI-Ia* was induced by both stresses in MM and TR154, but only by mite infestation in TR126 (**Figure 4F**). However, the JA-dependent *MYC-2* and PPO-F genes were not significantly differentially expressed in MM or TR accessions (**Figures 4C,E**).

Tomato plant defense proteins were affected by drought stress and mite infestation, but different responses were obtained depending on the cultivar/accessions (**Table 2** and **Supplementary Tables S6–S8**). In the case of MM, drought stress induced an increase in POD activity, but not when combined with mite infestation, and *T. evansi* infestation induced the inhibition activity against papain, cathepsin D, and chymotrypsin. TR154 showed a similar response: drought induced a decrease on the cathepsin B inhibitory activity; and *T. evansi* induced the inhibition of cathepsins B and D and papain. The accession TR126 showed the highest changes in the levels of defense proteins in response to stress: drought induced the inhibitory activity against cathepsin D, trypsin, and aminopeptidase; and *T. evansi* increased the inhibition against cathepsin B and chymotrypsin and reduced the inhibition against aminopeptidase.

**Table 2:** Effect of moderate drought, *T. evansi* infestation, and their combination (Dr + Te) on plant defense proteins of the tomato accessions TR126 and TR154 and the cultivar Moneymaker at 4 days post infestation.

	TR126					TR154					Moneymaker				
	Control	Drought	*T. evansi*	Dr + Te	SF	Control	Drought	*T. evansi*	Dr + Te	SF	Control	Drought	*T. evansi*	Dr + Te	SF
Protease inhibitors (% inhibition)															
Cathepsin B	47 ± 3	41 ± 2	55 ± 3	53 ± 3	M	46 ± 4	36 ± 2	58 ± 2	53 ± 3	D,M	42 ± 4	38 ± 5	54 ± 4	45 ± 5	–
Papain	66 ± 5	46 ± 7	71 ± 5	61 ± 11	–	49 ± 5	40 ± 6	70 ± 7	61 ± 11	M	50 ± 8	45 ± 9	72 ± 7	64 ± 5	M
Cathepsin D	36 ± 3	55 ± 4	47 ± 3	59 ± 5	D	47 ± 3	47 ± 5	65 ± 4	59 ± 5	M	45 ± 4	55 ± 5	67 ± 6	61 ± 5	M
Trypsin	24 ± 1	32 ± 2	27 ± 1	33 ± 2	D	36 ± 5	31 ± 1	36 ± 2	33 ± 2	–	32 ± 5	29 ± 3	36 ± 4	29 ± 4	–
Chymotrypsin	33 ± 5	35 ± 3	52 ± 5	49 ± 5	M	44 ± 3	38 ± 3	51 ± 5	49 ± 5	–	40 ± 2	29 ± 5	49 ± 5	43 ± 3	M
Aminopeptidase	36 ± 2	42 ± 1	34 ± 2	36 ± 1	D,M	31 ± 2	39 ± 6	41 ± 1	36 ± 1	–	37 ± 3	36 ± 3	43 ± 5	41 ± 3	–
Oxidative enzymes (specific activity)															
Polyphenol oxidases^1^	5.8 ± 0.7	7.4 ± 1.0	5.2 ± 0.7	5.1 ± 0.7	–	4.8 ± 0.6	5.5 ± 0.7	6.2 ± 1.0	5.1 ± 0.6	–	4.8 ± 0.5	6.2 ± 0.4	5.4 ± 0.7	5.0 ± 0.5	–
Peroxidases^2^	3.5 ± 0.4	3.2 ± 0.5	3.3 ± 0.4	3.6 ± 0.5	–	2.9 ± 0.3	3.8 ± 0.3	4.0 ± 0.6	4.0 ± 0.2	–	2.4 ± 0.3a	3.6 ± 0.4b	2.9 ± 0.3ab	2.8 ± 0.3ab	I


*Data are mean ± SE of 6 replicates/treatment. Significant factors (SF) indicates whether either of the two independent factors D (drought condition) and M (mite infestation) and/or their interaction I (D × M) are statistically significant for a given cultivar/accession (two-way ANOVA, *p* < 0.05). When the interaction D × M was significant, Newman-Keuls *post hoc* test were performed (different lowercase letters indicate significant differences among treatments). The detailed results of the two-way ANOVA (*F* and *p* values and degrees of freedom) are shown in **Supplementary Tables S6–S8**. ^*1*^PPO: nmol cathecol metabolized/mg protein^∗^min. ^*2*^POD: nmol guaiacol metabolized/mg protein^∗^min.*

### Effect of Sugars, Amino Acids, and L-Proline Infiltration on *T. evansi* Performance

**FIGURE 5:**
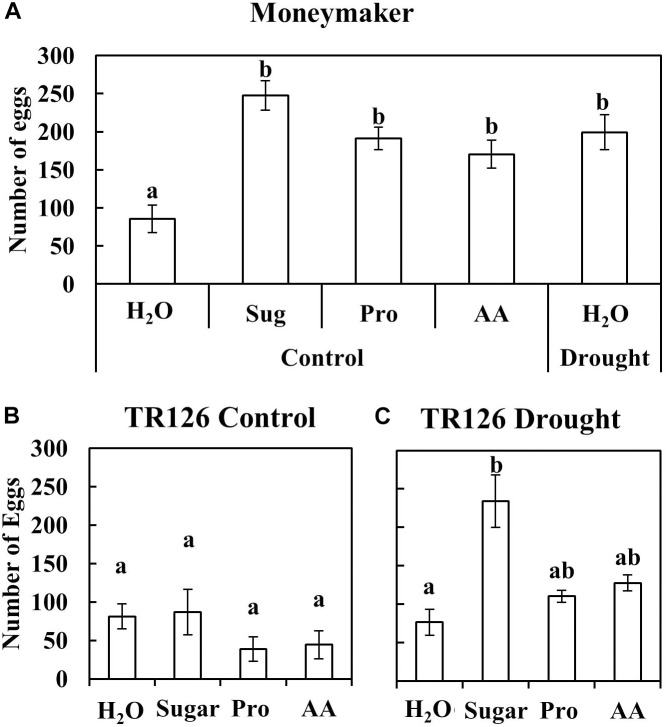
Number of eggs laid by *T. evansi* on leaflets from well-watered (Control) and drought-stressed (Drought) Moneymaker **(A)**, TR126 (Control) **(B)**, and TR126 (Drought) **(C)** tomato plants infiltrated with water (H_2_O), free sugars (Sug), amino acids (AA), and L-proline (Pro). Data are mean ± SE of five replicates/treatment at 24 h post infiltration. Different lowercase letters indicate significant differences among treatments (one-way ANOVA, Dunnett *post hoc* test, *p* < 0.05. **(A)** Moneymaker: *F*_4,20_: 9.52, *p* < 0.001; **(B)** control TR126: *F*_3,16_: 1.22, *p*: 0.335; **(C)** drought-stressed TR126: *F*_3,15_: 5.94, *p*: 0.007).

In order to test if the accumulation of nutrients in plants by drought stress is responsible for the enhanced mite performance, plant leaflets were infiltrated with free sugars and amino acids and used to fed mites. In the case of MM, the number of eggs laid by *T. evansi* on leaflets from well-watered (control) plants and infiltrated with free sugars, essential amino acids and L-proline was significantly higher than on leaflets from the same plants infiltrated with water, and similar to those laid on leaflets from drought-stressed plants and infiltrated with water (**Figure 5A**). We checked the amount of free sugars and L-proline in leaflets infiltrated with each of these nutrients, and their levels were significantly higher than on leaflets from control and drought-stressed plants infiltrated with water for at least 24 h, when the number of eggs was recorded (**Supplementary Figures S3A,B**). Interestingly, a significant correlation was found between the number of eggs laid and the levels of sugars (Pearson’s correlation coefficient (*r*): 0.678; *p*: 0.005) and L-proline (*r*: 0.608; *p*: 0.016) in the leaflets at 24 h post infiltration (**Supplementary Figures S3C,D**). When the accession TR126 was tested, no significant differences were observed between control plants infiltrated with free sugars, essential amino acids or L-proline, and the water-infiltrated controls (**Figure 5B**). Thus, the experiment was repeated using drought-stressed plants, and in this case, a significant increase in the number of eggs was obtained only with sugar-infiltrated plants (**Figure 5C**).

## Discussion

Our data reveal that drought stress has a differential effect on the performance of *T. evansi* in drought-adapted “Tomàtiga de Ramellet” tomatoes. Thus, the changes induced by drought stress on two of the TR accessions (TR61 and TR154) triggered a bottom up effect on *T. evansi*, increasing mite performance and the damage to the plant, following a similar pattern to that reported for the tomato cultivar MM (Ximénez-Embún et al., 2016). By contrast, no increase in mite performance was observed in TR58 and TR126, since no differences in the number of eggs laid and leaf damage were found between drought-stressed and control tomato plants. Drought stress has been reported to be positive, negative, or to have no effect on mite performance, depending on the mite species, the host plant, and the stress level (see Ximénez-Embún, 2017 for a review). However, in the case of tomato, the effect of drought conditions has been reported to be positive for three different mite species: *T. evansi* (Ximénez-Embún et al., 2016), *T. urticae* (Ximénez-Embún et al., 2017a), and *Aculops lycopersici* (Gispert et al., 1989; Ximénez-Embún et al., 2017b). This could have significant implications for mite outbreaks under future climate change scenarios, when longer periods of drought and less water availability are expected for irrigated crops such as tomato in semiarid environments. The finding that some accessions (such as TR126) were not more susceptible under stress conditions offers alternatives for pest management and provides an experimental framework for screening for drought-tolerant tomato accessions that are also resistant to herbivore mites.

A link could be observed between the nutritional composition of MM and the two accessions analyzed (TR154 and TR126) and *T. evansi* performance on these plants. Tomato plants have been reported to metabolize protein and starch into amino acids and free sugars, respectively, in response to drought stress (Bauer et al., 1997; Khodakovskaya et al., 2010; Ximénez-Embún et al., 2016). We have recorded an increase in the levels of free sugars and most essential amino acids for mites in MM (eight of a total of nine) and of most essential amino acids in TR154 (seven of nine) in response to drought stress, associated with a better performance of *T. evansi* on these plants. However, sugars were not altered and a comparatively reduced number of essential amino acids (four of nine) were induced by drought in TR126, an accession in which mite proliferation was not enhanced by drought stress. The positive effect of essential amino acids concentration on mite performance has been previously observed in *T. evansi* (Ximénez-Embún et al., 2016) and *T. urticae* (Tulisalo, 1971; Dabrowski and Bielak, 1978; Ximénez-Embún et al., 2017a). A special mention should be made to L-proline, traditionally used as a good indicator of plant response to drought (Claussen, 2005). We have found that this non-essential amino acid was induced in both TR126 and TR154, as well as in MM, indicating that the induction of L-proline alone is not sufficient for explaining the observed increase in the performance of *T. evansi*. Remarkably, we have shown that free sugars, L-proline, and essential amino acids had a stimulatory effect on *T. evansi* performance, when supplemented to control tomato MM leaflets, similar to that obtained in plants subjected to drought, supporting their role in enhancing mite performance as already reported for L-proline (Ximénez-Embún et al., 2016). However, only free sugars improved mite performance in TR126 when infiltrated into drought-stressed plants, whereas no stimulatory effect was observed when infiltrated in non-stressed plants. These results suggest that free sugars have a stronger stimulatory effect than L-proline and essential amino acids or that the response of the mites to these different nutrients depends on the tomato genetic background. Taken together, our results indicate that the accumulation of plants nutrients appears to play a key role in the higher suitability of drought-stressed tomato plants for *T. evansi*, though the contribution of L-proline, essential amino acids, and free sugars may vary among tomato genotypes. However, the fact that the infiltration of nutrients did not increase *T. evansi* performance under well-watered conditions in TR126 suggest that other factors can be involved. Thus, additional studies will be necessary to explain the mechanisms behind this association.

Plant defense is an important issue to be considered when assessing the plant response to biotic and abiotic stresses. We have investigated plant defense at three levels: at the phytohormone level, at the marker gene expression level and at the defense-protein activity level. The levels of defense-related phytohormones (OPDA, JA, SA, and SAGE) and the expression of SA-dependent (*PR1a*) and JA-dependent (*MYC2* and *PPO-F*) genes were not induced in MM after *T. evansi* infestation. This is in line with the reported suppression of plant defenses by *T. evansi* in tomato (Kant et al., 2015), including the downregulation of phytohormones and most of the defense genes analyzed (Sarmento et al., 2011; Alba et al., 2015; Ataide et al., 2016; Schimmel et al., 2017). However, we have found that mite infestation induced the expression of *CDI* and *PI-Ia* genes, which encodes for protease inhibitors, and increased cathepsin D, chymotrypsin, and papain inhibitory activities in MM. Thus, our results with MM corroborate the downregulation of defense-related phytohormones by *T. evansi*, but show that some defense genes can still be induced and plat defenses are not fully suppressed. Indeed, the induction of protease inhibitory activities in MM by *T. evansi* infestation has been previously reported (Ximénez-Embún et al., 2016). The ability of *T. evansi* to suppress the induction of defense-related phytohormones was also observed in the two TR accessions, with the exception of an increase in SA levels in TR126, though SAGE levels were not altered. The expression of the *CDI* and *PI-Ia* genes was also induced by *T. evansi* in both TR126 and TR154, as well as *PR1a* in TR154 and some of the protease inhibitory activities (cathepsin B and chymotrypsin in TR126 and cathepsin B, papain, and cathepsin D in TR154). This may have implications for tomato-mite interactions, since PIs are recognized as key components of the defensive response of tomato to mite infestation (Kant et al., 2004; Santamaria et al., 2012, 2015; Alba et al., 2015). *T. evansi* relies mostly on cysteine (cathepsin B, and L- and legumain-like) and aspartyl (cathepsin D-like) proteases and aminopeptidases for proteolytic digestion (Ximénez-Embún et al., 2016). Thus, the ingestion of PIs targeting some of these proteases may be harmful, as already demonstrated for *T. urticae* (Carrillo et al., 2011; Santamaria et al., 2012). Serine proteases do not appear to be directly involved in the hydrolysis of dietary proteins in this species, but tomato serine PIs may target other physiological processes, as has been indicated for *T. urticae* which has a similar digestive proteolytic profile (Santamaria et al., 2012, 2015). Since differences in the induction of protein defense genes and activities were found among the different plant genotypes tested, it will be of interest for the control of this invasive mite species to broaden the range of tested commercial and wild tomato cultivars, including other drought-adapted tomato accessions and varieties.

Our results suggest an association between ABA induction and the changes triggered by drought stress in MM and TR154 tomato plants, causing an increase in mite performance. Thus, ABA levels were significantly increased in MM and TR154 tomato plants by drought stress, but not in TR126. It is known that ABA lead to the accumulation of free amino acids, including L-proline, and to the conversion of starch to maltose, though subsequent free sugars accumulation appears to be regulated in an ABA-independent manner (Krasensky and Jonak, 2012). In addition, free sugars such as glucose modulate vital processes that are controlled by plant stress hormones and have been involved in the control of ABA biosynthesis and signaling, exemplifying the complex interplay of sugar and hormone signaling (Cheng et al., 2002; León and Sheen, 2003). However, the observed differences may also result from differential time responses to the ABA accumulation and signaling pathway, since the ABA-responsive *RAB18* gene was induced in all cultivar/accessions in response to drought stress. Moreover, ABA may modulate plant responses to mite infestation since it has been involved in the response to several biotic stresses with a positive or negative effect on the plant responses depending of the biotic stress (de Torres-Zabala et al., 2007; Ton et al., 2009). Further studies will be required to confirm the contribution of ABA in enhancing mite performance under drought conditions.

Salicylic acid and jasmonate, the two main regulators of biotic stress responses, have been also reported to be involved in the plant response to drought, interacting at different levels with the ABA signaling pathway on tomato plants (Muñoz-Espinoza et al., 2015; De Ollas and Dodd, 2016). However, our results suggest that the changes operated in MM and TR154 tomato plants by drought stress, causing an increase in mite performance, are independent of SA and JA signaling. The SA signaling pathway regulates the expression of a large set of defense-related genes, including pathogen-related proteins (PRs), and has been involved in tomato response defense to mites (Glas et al., 2014; Alba et al., 2015), though the mechanism of action is not clearly understood. We have found that the levels of SA and SAGE on TR126 and TR154 plants were threefold to fivefold lower than on MM under control conditions. However, SA levels were strongly induced by drought in both TR accessions, whereas drought induced SAGE levels in TR154 and reduced them in TR126. On the contrary, the levels of these two hormones were not altered by drought stress in MM, as previously reported (Ximénez-Embún et al., 2017b). In addition, the expression of the SA-dependent *PR1a* gene was induced by drought in TR126 and by mite infestation in TR154, but not in MM. The observed differences between the two TR accessions and MM may be related to their distinct antioxidant status during control and/or drought stress conditions, since SA has been reported to mediate the response to drought stress providing protection against oxidative damage in tomato plants (Hayat et al., 2008). The JA pathway has been reported to play a key role in tomato plant resistance to mites, mediated by the induction of plant defense marker genes and proteins (Glas et al., 2014; Alba et al., 2015). However, our results suggest a complex regulation of the JA pathway in the two TR accessions and MM in response to drought stress. We found that drought stress reduced the levels of the JA precursor OPDA in MM and TR126 and that significantly lower JA levels occurred on uninfested drought-stressed TR126 plants. However, the *CDI* gene was induced by drought in all tomato cultivar/accessions tested, and the *PI-Ia* gene by drought in MM and when both stresses were combined in TR154. Moreover, trypsin, cathepsin D, and aminopeptidase inhibitory activities were induced in TR126 by drought stress, cathepsin B inhibitory activity was reduced by drought stress in TR154, and POD activity was induced in uninfested drought-stressed MM plants. The potential implications of the ingestion of the induced PIs in mite performance have been discussed above. With regard to antioxidant enzymes, POD activity has been reported to be induced in tomato by drought stress (English-Loeb et al., 1997; Inbar et al., 2001). The production of PODs is an adaptive mechanism for the scavenging and detoxification of reactive oxygen species in drought-stressed plants (Rai et al., 2013), but its induction might vary depending on the tomato plant genotype (Sanchez-Rodriguez et al., 2010). This might explain the absence of induction in the case of TR126 and TR154 plants.

Altogether, our data revealed differential plant-mediated effects of water deficit on the performance of *T. evansi* on drought-adapted “Tomàtiga de Ramellet” tomatoes. These findings have important implications for decision making in the selection of tomato cultivars to be planted in a forthcoming climate change scenario, as their differential response to water deficit might speed up or slow down the expansion of this important invasive mite species in extensive tomato production. In addition, the plant-mediated effects reported here may have ecological implications, since it is known that the suppression of tomato plant defenses by *T. evansi* have effects on its competition with other spider mites (Oliveira et al., 2016).

## Author Contributions

PC, FO, MX-E, and MG-G conceived and designed the experiments. MX-E and MG-G performed the experiments. VA and AG-C performed the phytohormone experiments. PC, FO, MX-E MG-G, VA, and AG-C analyzed the data. PC, FO, MG-G, VA, and AG-C contributed reagents, materials, and analysis tools. PC, FO, MX-E, and MG-G wrote the paper. All authors revised the final version of the manuscript.

## Conflict of Interest Statement

The authors declare that the research was conducted in the absence of any commercial or financial relationships that could be construed as a potential conflict of interest. The handling Editor declared a shared department, though no other collaboration with three of the authors VA, AG-C, and MG-G.

## Footnotes

1 www.gimp.org
