# Supplementary material for: Plant-Mediated Effects of Water Deficit on the Performance of Tetranychus evansi on Tomato Drought-Adapted Accessions
Source: Front Plant Sci. 2018 Oct 17;9:1490. doi: 10.3389/fpls.2018.01490 (PMC6199365; doi:10.3389/fpls.2018.01490)
Supplement: Supplementary file 1 [file Data_Sheet_1.pdf]

Supplementary Material

Plant-mediated effects of water deficit on the performance of *Tetranychus evansi* on tomato drought-adapted accessions

Miguel G. Ximénez-Embún, Miguel Gonzalez-Guzman, Vicent Arbona, Aurelio Gómez-Cadenas, Félix Ortego, Pedro Castañera*

*** Correspondence:** Pedro Castañera castan@cib.csic.es

1. Supplementary Figures

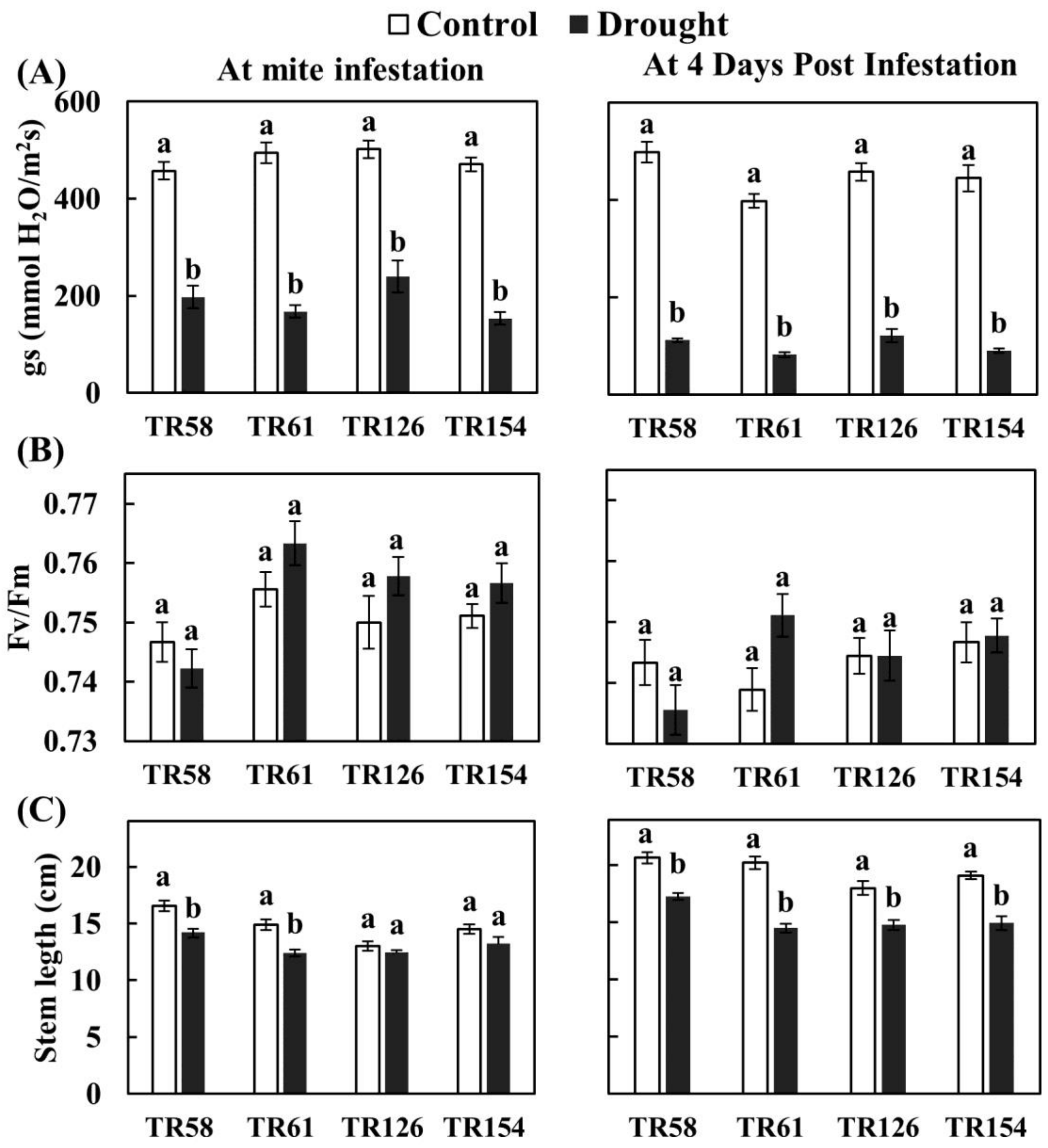

Supplementary Figure 1. Effect of moderate drought on A) stomatal conductance (gs), B) maximum quantum yield of PSII photochemistry (Fv/Fm) and C) stem length of the tomato accessions TR58, TR61, TR126 and TR154 at mite infestation and at 4 days post infestation. Data shown are mean ± SE of 9 replicates/treatment from Experiment 1. Different lowercase letters indicate significant differences within each accession (Student's t-test, $p<0.05$). The detailed results (t and p values and degrees of freedom) are shown in Supplementary Table 3.

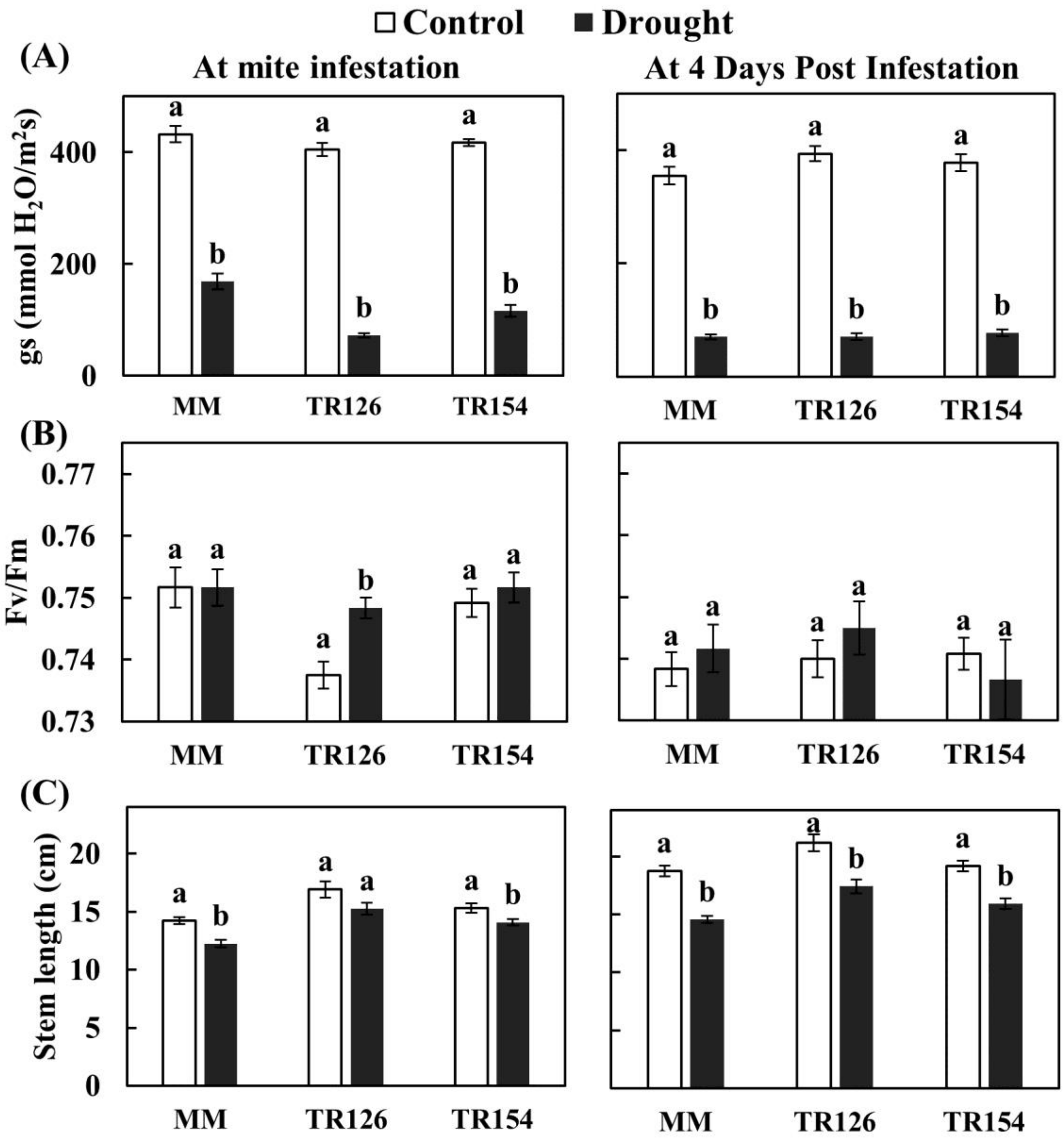

Supplementary Figure 2. Effect of moderate drought on A) stomatal conductance (gs), B) maximum quantum yield of PSII photochemistry (Fv/Fm) and C) stem length of the tomato cultivar Moneymaker (MM) and the accessions TR126 and TR154 at mite infestation and at 4 days post infestation. Data (mean ± SE) shown are from infested (6 replicates) plus non-infested (6 replicas) plants on Experiment 2. Different lowercase letters indicate significant differences within each cultivar/accession (Student's t-test, $p<0.05$). The detailed results (t and p values and degrees of freedom) are shown in Supplementary Table 4.

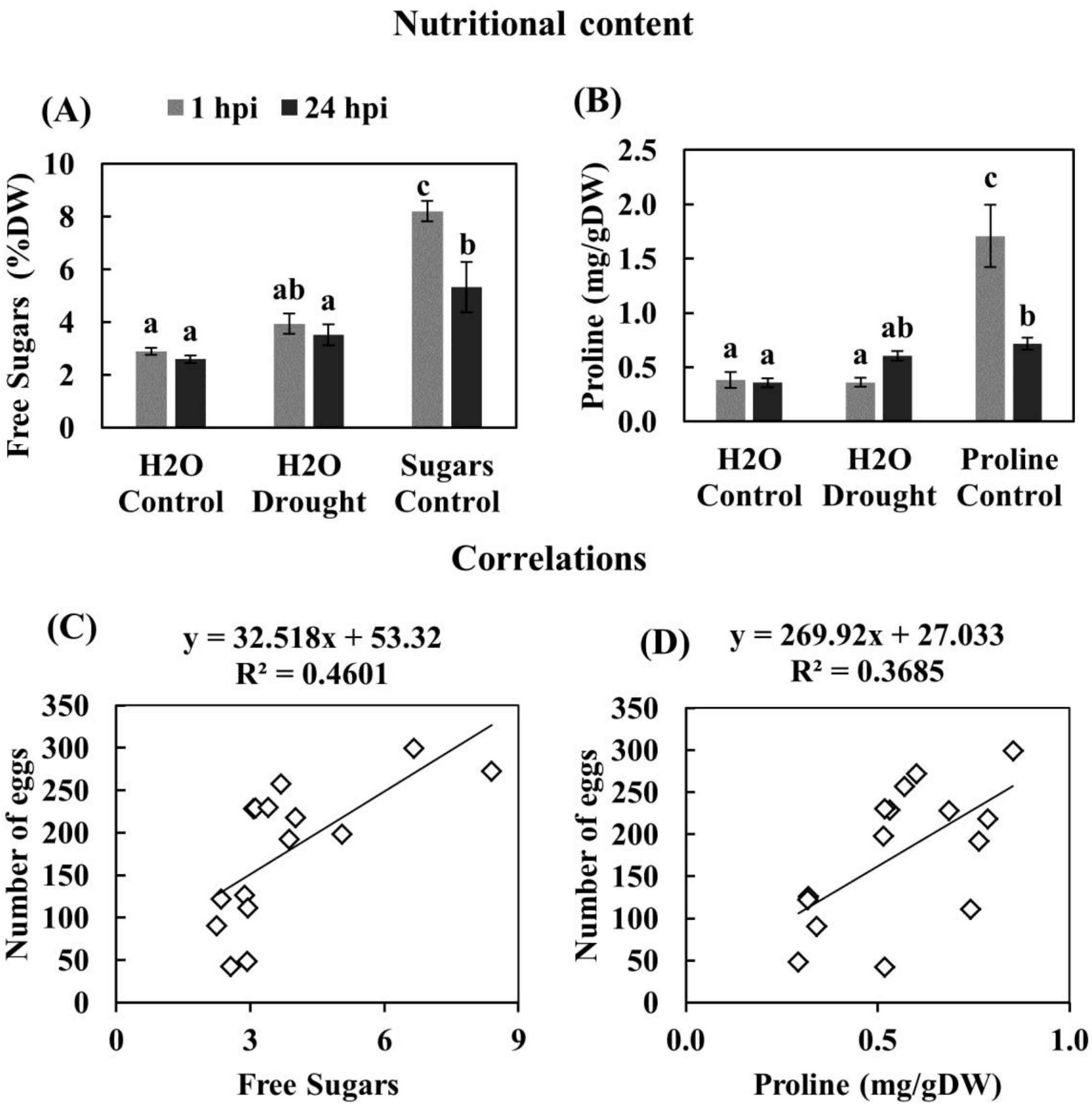

Supplementary Figure 3. Content of free sugars (A) and L-proline (B) of control tomato Moneymaker leaflets infiltrated with water, sugar or proline and of drought stressed Moneymaker leaflets infiltrated with water. Data are mean±S.E. of five replicates/treatment at 1 hour post infiltration (hpi) and 24 hpi. Different lowercase letters indicate significant differences among all combinations of treatments and time (One-way ANOVA, Newman-Keuls post hoc test, $p<0.05$. A) Free sugars: $F_{1,24}$: 19.72, $p<0.001$; B) L-Proline $F_{1,24}$: 24.76, $p<0.001$]). Correlation between the leaf content of free sugars (C) and L-proline (D) and the number of eggs laid by *T. evansi* in infiltrated plants at 24 hpi. The Pearson´s correlation coefficient (r) was significant for both free sugars (r: 0.678; p: 0.005) and L-proline (r: 0.608; p: 0.016).

1 Supplementary Tables

Supplementary Table 1. Summary of analytical methods used to assess the inhibitory activity of plant protein extracts[1]

Commercial enzyme [2]	Substrate [3]	Buffer [4]	Incubation	Measurement [5]
Cathepsin B from bovine spleen (EC 3.4.22.1)	Z-RR-AMC	100 mM NA phosphate, pH 6.0 (10 mM L-cysteine, 10 mM EDTA, 0.01% (v/v) Brij 35)	30 min at 28 ºC	excitation filter 350 nm emission filter 465 nm
Papain (EC 3.4.22.2)	Z-FR-AMC	100 mM Na phosphate, pH 6.0 (10 mM L-cysteine, 10 mM EDTA, 0.01% (v/v) Brij 35)	30 min at 28 ºC	excitation filter 350 nm emission filter 465 nm
Cathepsin D from bovine spleen (EC 3.4.23.5)	MocAc-GKPILFFRLK (Dnp)-D-R-NH2	100 mM sodium citrate, pH 3.5 (0.15M NaCl, 5 mM MgCl2)	20 min at 30 ºC	excitation filter 328 nm emission filter 393 nm
Trypsin from bovine pancreas (EC 3.4.21.4)	Z-LA-AMC	100 mM Tris-HCl, pH 7.5 (0.15M NaCl, 5 mM MgCl2)	1 h at 35 ºC	excitation filter 350 nm emission filter 465 nm
α-Chymotrypsin from bovine pancreas (EC 3.4.21.1)	SucAAPF-AMC	100 mM Tris-HCl, pH 7.5 (0.15M NaCl, 5 mM MgCl2)	30 min at 35 ºC	excitation filter 350 nm emission filter 465 nm
Leucine aminopeptidase from porcine pancreas (EC 3.4.11.1)	LpNa	100 mM Tris-HCl, pH 8 (0.15M NaCl, 5 mM MgCl2)	30 min at 30 ºC	absorbance at 410 nm

[1] Procedures adapted from Ximénez-Embún et al. (2016). Samples of 20 µg of plant protein extracts (40 µg in case of leucine aminopeptidase) were preincubated for 10 min with 100 ng of the commercial enzyme.

[2] All purchased from Sigma-Aldricht (St Luis, USA).

[3] The substrates were added at a final concentration of 20 µM. Z-RR-AMC (N-carbobenzoxyloxy-Arg-Arg-7-amido-4-methylcoumarin) for cathepsin B, Z-FR-AMC (N-carbobenzoxyloxy-Phe-Arg-7-amido-4-methylcoumarin) for papain, Z-LA-AMC (Z-L-Arg-7-amido-4-methylcoumarin) for trypsin, SucAAPF-AMC (Suc-Ala-Ala-Pro-Phe-7-amido-4-methylcoumarin) for chymotrypsin, all purchased from Calbiochem (MerkMilipore, Billerica, USA), MocAc-GKPILFFRLK(Dnp)-D-R-NH2 from Peptanova (Germany) for cathepsin D, and LpNa (L-leucine p-nitroanilide) from Sigma-Aldricht (St Luis, USA) for leucine aminopeptidase.

[4] Concentrations are expressed at molarity in the reaction mixture.

[5] AMC (7-amino-4-methylcoumarin) (Bachem, Swizerland) as standard for all fluorescent substrates, except MCA (MoCAC-Pro-Leu-Gly) (Peptanova GmbH, Germany) for cathepsin D. Double blanks were used to account for spontaneous breakdown of substrates and the plant protease activity, and all assays were done in duplicate.

Supplementary Table 2 Nucleotide sequence of primers used for qRT-PCR analysis

Gene	Name	Gen Model ITAG2,3	Forward Primer (5´→3´)	Reverse Primer (5´→3´)	Reference [1]
RAB18	Responsive to ABA 18	Solyc02g084850.2	CCTGGGATGCATTGAACACC	CACGGGACACCATAACACAC	Gonzalez-Guzman et al., 2014
PR-1a	Pathogenesis-related protein 1a	Solyc09g007010.1.1	TGGTGGTTCATTTCTTGCAACTAC	ATCAATCCGATCCACTTATCATTTTA	Alba et al., 2015
MYC2		Solyc08g076930.1.1	CGGTGTCATCACCTGCTTAT	TTCGGTGTCGGTAACTTCTTC	This work
PPO-F	Polyphenol-oxidase-F	Solyc08g074630.1.1	CGGAGTTTGCAGGGAGTTATAC	TTGATCTCCACACTTTCAATGG	Alba et al., 2015
CDI	Cathepsin D inhibitor protein	Solyc03g098790.1.1	ACTCGTCCTGTGCTTTGTCC	CCCAAGAGGATTTTCGTTGA	Lisón et al., 2006
PI-Ia	Protease Inhibitor Ia	Solyc09g084470	TGTACAAATGCCTGTGGTGACT	GGAGTACATGTAATTAAGCCACACT	Martel et al., 2015
Actin		Solyc03g078400.2.1	CCTCAGCACATTCCAGCAG	CCACCAAACTTCTCCATCCC	Martel et al., 2015

[1] See References list in the main text.

González-Guzmán et al. (2014). J. Exp. Bot. 65(15), 4451-4464. doi: 10.1093/jxb/eru219
Lisón et al. (2006) *Plant Physiol* 142(3), 1329–1339. doi: 10.1104/pp.106.086587
Martel et al. (2015) *Mol Plant Microbe Interact* 28(3), 343-61. doi: 10.1094/MPMI-09-14-0291-FI.

Supplementary Table 3. Results of the Student's t-test of stomatal conductance, Fv/Fm and stem length for each accession at each time point in Experiment 1.

	TR58			TR61			TR126			TR154		
	t	df	p	t	df	p	t	df	p	t	df	p
**At mite infestation**												
**Stomatal conductance**	7.980	16	**<0.001**	11.645	16	**<0.001**	5.650	16	**<0.001**	12.224	16	**<0.001**
**Fv/Fm**	0.956	16	0.353	-1.635	16	0.122	-1.419	16	0.175	-1.425	16	0.173
**Stem length**	4.180	16	**0.001**	4.180	16	**0.001**	1.170	16	0.259	1.785	16	0.093
**At 4 days post infestation**												
**Stomatal conductance**	26.059	16	**<0.001**	21.675	16	**<0.001**	11.194	16	**<0.001**	19.954	16	**<0.001**
**Fv/Fm**	1.400	16	0.181	1.400	16	0.181	0.004	16	0.997	-0.258	16	0.800
**Stem length**	5.921	16	**<0.001**	8.612	16	**<0.001**	4.319	16	**<0.001**	5.339	16	**<0.001**

Supplementary Table 4. Results of the Student's t-test of stomatal conductance, Fv/Fm and stem length for each cultivar/accession at each time point in Experiment 2.

	Moneymaker			TR126			TR154		
	t	df	p	t	df	p	t	df	p
**At mite infestation**									
**Stomatal conductance**	9.884	22	**<0.001**	27.143	22	**<0.001**	15.302	22	**<0.001**
**Fv/Fm**	-0.001	22	0.999	-3.953	22	**0.001**	-0.751	22	0.461
**Stem length**	4.446	22	**<0.001**	1.953	22	0.064	2.480	22	**0.021**
**At 4 days post infestation**									
**Stomatal conductance**	20.870	22	**<0.001**	18.902	22	**<0.001**	18.621	22	**<0.001**
**Fv/Fm**	-0.701	22	0.490	-0.935	22	0.360	0.608	22	0.549
**Stem length**	8.041	22	**<0.001**	4.011	22	**0.001**	4.980	22	**<0.001**

Supplementary Table 5. Results of the Student's t-test of number of eggs, mobile forms and leaf damage for each accession.

	TR58			TR61			TR126			TR154		
	t	df	p	t	df	p	t	df	p	t	df	p
**Number of eggs**	0.233	16	0.819	-2.541	16	**0.022**	0.895	16	0.384	-3.603	16	**0.002**
**Mobile forms**	-0.383	16	0.707	-1.237	16	0.234	0.011	16	0.991	-1.683	16	0.112
**Leaf damage**	-0.714	16	0.486	-2.558	16	**0.021**	0.493	16	0.629	-4.069	16	**0.001**

Supplementary Table 6 Results of the two-way ANOVA analysis of Accession TR126.

	**Drought**			***T. evansi***			**Drought*T.evansi**		
	**F**	**df**	**p**	**F**	**df**	**p**	**F**	**df**	**p**
**Nutrients**									
**Water**	0.034	1,20	0.857	0.286	1,20	0.599	1.338	1,20	0.261
**Free sugars**	3.976	1,20	0.060	0.326	1,20	0.575	1.062	1,20	0.315
**Protein**	0.689	1,20	0.416	0.449	1,20	0.596	0.862	1,20	0.364
**Total free aa**	2.780	1,20	0.111	0.140	1,20	0.712	1.395	1,20	0.251
**Non-essential amino acids**									
**Asp**	0.178	1,20	0.677	0.027	1,20	0.870	2.114	1,20	0.161
**Thr**	4.458	1,20	**0.048**	0.009	1,20	0.924	1.366	1,20	0.256
**Ser**	0.393	1,20	0.538	0.530	1,20	0.475	0.136	1,20	0.716
**Glu**	5.041	1,20	**0.036**	0.497	1,20	0.489	4.836	1,20	**0.040**
**Gly**	1.814	1,20	0.193	0.881	1,20	0.359	0.116	1,20	0.737
**Ala**	0.102	1,20	0.752	0.300	1,20	0.590	0.054	1,20	0.819
**Cys**	0.983	1,20	0.333	0.425	1,20	0.522	0.339	1,20	0.567
**Pro**	28.56	1,20	**<0.001**	0.224	1,20	0.641	0.092	1,20	0.765
**Essential amino acids**									
**Val**	6.656	1,20	**0.018**	1.519	1,20	0.232	0.089	1,20	0.769
**Met**	0.126	1,20	0.726	3.267	1,20	0.086	1.146	1,20	0.297
**Ile**	5.355	1,20	**0.031**	2.747	1,20	0.113	0.197	1,20	0.662
**Leu**	4.511	1,20	**0.046**	2.957	1,20	0.101	1.050	1,20	0.318
**Tyr**	3.114	1,20	0.093	3.608	1,20	0.072	0.865	1,20	0.363
**Phe**	4.671	1,20	**0.043**	1.803	1,20	0.194	0.088	1,20	0.769
**His**	2.400	1,20	0.137	3.023	1,20	0.097	0.002	1,20	0.964
**Lys**	1.664	1,20	0.212	2.869	1,20	0.106	0.309	1,20	0.584
**Arg**	2.398	1,20	0.137	1.453	1,20	0.242	0.042	1,20	0.840
**Phytohormones**									
**ABA**	1.794	1,16	0.199	0.472	1,16	0.502	0.791	1,16	0.387
**JA**	4.706	1,16	**0.045**	1.375	1,16	0.258	5.998	1,16	**0.026**
**OPDA**	12.59	1,16	**0.003**	0.055	1,16	0.818	0.042	1,16	0.840
**SA**	4.561	1,16	**0.049**	15.33	1,16	**0.001**	0.001	1,16	0.972
**SAGE**	122.8	1,16	**<0.001**	0.628	1,16	0.440	0.502	1,16	0.489
**Gene expression**									
RAB-18	17.32	1,20	**<0.001**	0.435	1,20	0.517	30.99	1,20	**<0.001**
PR1a	68.37	1,20	**<0.001**	1.195	1,20	0.287	4.315	1,20	0.051
MYC-2	2.045	1,20	0.168	0.082	1,20	0.778	0.348	1,20	0.562
CDI	7.920	1,20	**0.011**	38.11	1,20	**<0.001**	1.908	1,20	0.182
PPO-F	3.181	1,20	0.090	0.043	1,20	0.838	2.395	1,20	0.137
PI-Ia	0.341	1,20	0.566	5.343	1,20	**0.032**	0.032	1,20	0.859
**Defense proteins**									
**Cathepsin B**	2.823	1,20	0.108	13.68	1,20	**0.001**	0.490	1,20	0.492
**Papain**	3.374	1,20	0.081	1.861	1,20	0.188	0.168	1,20	0.686
**Cathepsin D**	7.802	1,20	**0.011**	1.584	1,20	0.223	0.390	1,20	0.539
**Trypsin**	11.74	1,20	**0.003**	0.481	1,20	0.496	0.259	1,20	0.616
**Chymotrypsin**	0.026	1,20	0.873	14.31	1,20	**0.001**	0.476	1,20	0.498
**Aminopeptidase**	7.612	1,20	**0.012**	5.894	1,20	**0.025**	1.701	1,20	0.207
**Polyphenol oxidases**	0.722	1,20	0.406	3.107	1,20	0.093	0.598	1,20	0.448
**Peroxidases**	0.029	1,20	0.865	0.029	1,20	0.865	0.490	1,20	0.492
	**F**	**df**	**p**	**F**	**df**	**p**	**F**	**df**	**p**
	**Drought**			***T. evansi***			**Drought*T.evansi**		

Supplementary Table 7 Results of the two-way ANOVA analysis of Accession TR154.

	Drought			*T. evansi*			Drought**T.evansi*		
	**F**	**df**	**p**	**F**	**df**	**p**	**F**	**df**	**p**
**Nutrients**									
**Water**	2.722	1,20	0.115	0.08	1,20	0.780	5.197	1,20	**0.034**
**Free sugars**	2.49	1,20	0.131	12.82	1,20	**0.002**	5.713	1,20	**0.027**
**Protein**	3.546	1,20	0.074	1.472	1,20	0.239	16.47	1,20	**<0.001**
**Total free aa**	2.174	1,20	0.156	0.735	1,20	0.401	4.023	1,20	0.059
**Non-essential amino acids**									
**Asp**	0.222	1,20	0.643	0.064	1,20	0.802	4.063	1,20	0.057
**Thr**	1.498	1,20	0.235	0.104	1,20	0.750	3.517	1,20	0.075
**Ser**	12.35	1,20	**0.002**	1.234	1,20	0.280	1.824	1,20	0.192
**Glu**	0.048	1,20	0.829	0.446	1,20	0.512	5.081	1,20	**0.036**
**Gly**	0.018	1,20	0.895	0.026	1,20	0.873	2.741	1,20	0.113
**Ala**	0.133	1,20	0.719	0.157	1,20	0.696	4.006	1,20	0.059
**Cys**	1.740	1,20	0.202	0.964	1,20	0.338	4.343	1,20	0.050
**Pro**	30.20	1,20	**<0.001**	1.018	1,20	0.325	3.268	1,20	0.086
**Essential amino acids**									
**Val**	10.75	1,20	**0.004**	2.043	1,20	0.168	1.415	1,20	0.248
**Met**	0.264	1,20	0.613	0.010	1,20	0.920	2.542	1,20	0.127
**Ile**	10.79	1,20	**0.004**	2.359	1,20	0.140	0.803	1,20	0.381
**Leu**	9.280	1,20	**0.006**	2.478	1,20	0.131	0.936	1,20	0.345
**Tyr**	10.10	1,20	**0.005**	2.672	1,20	0.118	0.160	1,20	0.694
**Phe**	2.367	1,20	0.140	1.475	1,20	0.239	2.123	1,20	0.161
**His**	16.22	1,20	**0.001**	4.313	1,20	0.051	0.980	1,20	0.334
**Lys**	17.19	1,20	**<0.001**	2.682	1,20	0.117	1.882	1,20	0.185
**Arg**	7.525	1,20	**0.013**	2.090	1,20	0.164	0.199	1,20	0.660
**Phytohormones**									
**ABA**	64.01	1,18	**<0.001**	0.938	1,18	0.346	4.868	1,18	**0.041**
**JA**	0.454	1,18	0.509	0.946	1,18	0.344	3.903	1,18	0.064
**OPDA**	2.968	1,18	0.102	1.855	1,18	0.190	4.578	1,18	**0.046**
**SA**	30.73	1,18	**<0.001**	1.972	1,18	0.177	11.36	1,18	**0.003**
**SAGE**	76.32	1,18	**<0.001**	37.52	1,18	**<0.001**	106.6	1,18	**<0.001**
**Gene expression**									
RAB-18	1.058	1,20	0.316	3.803	1,20	0.065	7.397	1,20	**0.013**
PR1a	2.457	1,20	0.133	4.346	1,20	**0.050**	0.138	1,20	0.714
MYC-2	0.212	1,20	0.651	0.763	1,20	0.394	3.112	1,20	0.094
CDI	10.91	1,20	**0.004**	18.79	1,20	**<0.001**	1.114	1,20	0.304
PPO-F	1.448	1,20	0.243	3.152	1,20	0.091	1.560	1,20	0.226
PI-Ia	9.222	1,20	**0.007**	7.486	1,20	**0.013**	5.173	1,20	**0.034**
**Defense proteins**									
**Cathepsin B**	7.347	1,20	**0.013**	25.06	1,20	**<0.001**	0.799	1,20	0.382
**Papain**	1.324	1,20	0.263	7.93	1,20	**0.011**	0.002	1,20	0.962
**Cathepsin D**	0.538	1,20	0.472	11.71	1,20	**0.003**	0.469	1,20	0.501
**Trypsin**	2.026	1,20	0.170	0.141	1,20	0.711	0.013	1,20	0.912
**Chymotrypsin**	1.182	1,20	0.290	4.228	1,20	0.053	0.265	1,20	0.613
**Aminopeptidase**	0.242	1,20	0.628	1.459	1,20	0.241	3.991	1,20	0.060
**Polyphenol oxidases**	0.028	1,20	0.868	0.315	1,20	0.581	1.390	1,20	0.252
**Peroxidases**	2.667	1,20	0.118	3.809	1,20	0.065	1.625	1,20	0.217
	**F**	**df**	**p**	**F**	**df**	**p**	**F**	**df**	**p**
	**Drought**			***T. evansi***			**Drought*T.evansi**		

Supplementary Table 8 Results of the two-way ANOVA analysis of Moneymaker.

	**Drought**			***T. evansi***			**Drought*T.evansi**		
	**F**	**df**	**p**	**F**	**df**	**p**	**F**	**df**	**p**
**Nutrients**									
**Water**	1.150	1,20	0.296	2.957	1,20	0.101	4.065	1,20	0.057
**Free sugars**	12.37	1,20	**0.002**	0.278	1,20	0.604	0.057	1,20	0.814
**Protein**	0.968	1,20	0.337	0.143	1,20	0.709	0.036	1,20	0.861
**Total free aa**	11.66	1,20	**0.003**	1.308	1,20	0.266	1.154	1,20	0.296
**Non-essential amino acids**									
**Asp**	3.539	1,20	0.075	0.199	1,20	0.660	0.570	1,20	0.459
**Thr**	9.003	1,20	**0.007**	0.391	1,20	0.539	0.213	1,20	0.649
**Ser**	10.17	1,20	**0.005**	0.717	1,20	0.407	0.668	1,20	0.423
**Glu**	4.194	1,20	**0.054**	6.272	1,20	**0.021**	5.116	1,20	**0.035**
**Gly**	3.403	1,20	0.080	0.044	1,20	0.835	0.030	1,20	0.865
**Ala**	2.631	1,20	0.120	0.018	1,20	0.894	0.002	1,20	0.962
**Cys**	4.890	1,20	**0.039**	0.034	1,20	0.855	0.022	1,20	0.885
**Pro**	16.38	1,20	**0.001**	0.865	1,20	0.364	0.850	1,20	0.368
**Essential amino acids**									
**Val**	7.496	1,20	**0.013**	0.362	1,20	0.554	0.150	1,20	0.703
**Met**	3.811	1,20	0.065	0.326	1,20	0.574	0.217	1,20	0.647
**Ile**	7.675	1,20	**0.012**	0.556	1,20	0.465	0.235	1,20	0.633
**Leu**	6.009	1,20	**0.024**	0.147	1,20	0.706	0.019	1,20	0.891
**Tyr**	7.087	1,20	**0.015**	0.641	1,20	0.433	0.246	1,20	0.625
**Phe**	5.322	1,20	**0.032**	0.155	1,20	0.698	0.062	1,20	0.806
**His**	10.03	1,20	**0.005**	1.428	1,20	0.246	0.636	1,20	0.434
**Lys**	6.754	1,20	**0.017**	0.114	1,20	0.739	0.016	1,20	0.899
**Arg**	9.306	1,20	**0.006**	0.778	1,20	0.388	0.517	1,20	0.480
**Phytohormones**									
**ABA**	37.75	1,19	**<0.001**	2.483	1,19	0.132	3.340	1,19	0.083
**JA**	2.571	1,19	0.125	0.004	1,19	0.953	0.015	1,19	0.905
**OPDA**	10.50	1,19	**0.004**	1.905	1,19	0.184	4.666	1,19	**0.044**
**SA**	0.263	1,19	0.614	0.309	1,19	0.585	0.069	1,19	0.795
**SAGE**	0.410	1,19	0.530	0.415	1,19	0.527	1.003	1,19	0.329
**Gene expression**									
RAB-18	28.79	1,18	**<0.001**	0.265	1,18	0.613	13.41	1,18	**0.002**
PR1a	0.484	1,18	0.496	0.154	1,18	0.699	0.162	1,18	0.692
MYC-2	1.601	1,17	0.223	0.383	1,17	0.544	0.057	1,17	0.814
CDI	20.79	1,17	**<0.001**	39.37	1,17	**<0.001**	0.029	1,17	0.867
PPO-F	2.243	1,18	0.152	4.018	1,18	0.060	3.088	1,18	0.096
PI-Ia	11.28	1,18	**0.003**	6.979	1,18	**0.017**	2.745	1,18	0.115
**Defense proteins**									
**Cathepsin B**	1.759	1,17	0.202	4.255	1,17	0.055	0.296	1,17	0.593
**Papain**	1.003	1,17	0.331	7.899	1,17	**0.012**	0.115	1,17	0.739
**Cathepsin D**	0.105	1,17	0.750	8.090	1,17	**0.011**	2.630	1,17	0.123
**Trypsin**	1.078	1,17	0.314	0.307	1,17	0.587	0.356	1,17	0.559
**Chymotrypsin**	4.866	1,17	0.041	8.000	1,17	**0.012**	0.426	1,17	0.523
**Aminopeptidase**	0.171	1,17	0.684	2.467	1,17	0.135	0.019	1,17	0.893
**Polyphenol oxidases**	0.982	1,20	0.334	0.265	1,20	0.612	2.904	1,20	0.104
**Peroxidases**	4.121	1,20	0.056	0.119	1,20	0.734	4.438	1,20	**0.048**
	**F**	**df**	**p**	**F**	**df**	**p**	**F**	**df**	**p**
	**Drought**			***T. evansi***			**Drought*T.evansi**		
